# Confined nanopores and hydrogen bonds of polyoxometalates for continuous electricity generation from fluctuating humidity

**DOI:** 10.1039/d6sc00287k

**Published:** 2026-04-01

**Authors:** Tuo Ji, WeiLin Chen, Fan Liao, ZhenHui Kang

**Affiliations:** a Key Laboratory of Polyoxometalate and Reticular Material Chemistry of Ministry of Education, Northeast Normal University Changchun 130024 P. R. China chenwl@nenu.edu.cn; b State Key Laboratory of Bioinspired Interfacial Materials Science, Institute of Functional Nano and Soft Materials (FUNSOM), Soochow University Suzhou 215123 P. R. China fliao@suda.edu.cn zhkang@suda.edu.cn; c Macao Institute of Materials Science and Engineering (MIMSE), MUST-SUDA Joint Research Center for Advanced Functional Materials, Macau University of Science and Technology Taipa 999078 Macao P. R. China zhkang@must.edu.mo

## Abstract

Environmental humidity power generation technology is an important strategy to reduce the use of fossil fuels and solve the energy crisis. However, it remains a challenge to maintain a water adsorption gradient for the long-term output of continuous electrical energy when environmental humidity fluctuates. Herein, porous polyoxometalate (POM) nanomaterial [Cu^II^(2,2′-bipy)(H_2_O)_2_Cl]_*n*_[Cu^II^-(2,2′-bipy)(H_2_O)_2_Al(OH)_6_Mo_6_O_18_]_*n*_ (Cu-CuAlMo_6_) was used to assemble thin-film devices, which achieved continuous power generation in fluctuating humidity. The device generated stable electrical output (0.203 V, 4 µA cm^−2^, maximum power density of 0.06 µW cm^−2^) in 10% humidity, and maintained continuous electrical output (0.246 V, 14.5 µA cm^−2^, maximum power density of 0.214 µW cm^−2^) in high humidity, even with condensed water for 8 days. A detectable electrical response was generated within 0.1 s under the humidity trigger and enabled real-time tracking of environmental and chemical information. First-principles calculations elucidated that hygroscopic sites constructed by the oxygen-containing groups and hydrogen bonds in the POM ensured efficient collection of humidity, and confined nanopores maintained the water adsorption gradient under high humidity. In addition, the unique charge transfer mechanism enabled the device to autonomously monitor environmental and chemical information in real-time. This work provides a reliable strategy for developing humidity power generation technology that continuously outputs in fluctuating environmental humidity, and is expected to form important components of multimodal real-time monitoring systems.

## Abstract 2

A continuous humidity power generation strategy based on polyoxometalates (POMs) with confined nanopores and hydrogen bonds for effectively collecting water molecules and maintaining water adsorption gradient in fluctuating environmental humidity.

## Graphical abstract

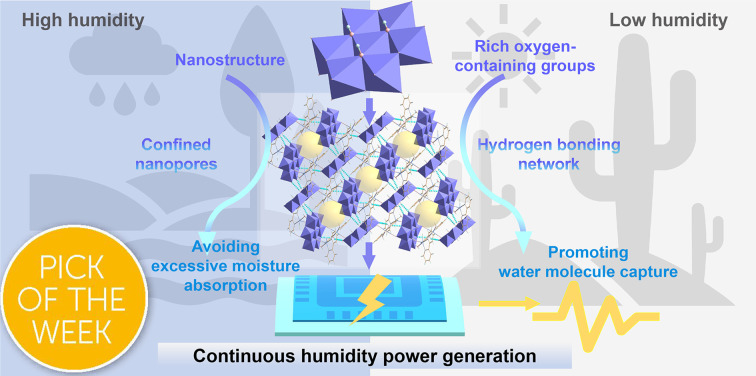

## Introduction

The development and utilization of sustainable clean energy are important strategies to reduce the consumption of fossil fuels, solve energy crises, and decrease environmental degradation.^1–3^ Water is a widely distributed resource with great potential for use as a clean and recyclable energy source on Earth.^4–7^ There are broad application prospects for humidity power generation technology because it utilizes the dynamic adsorption–desorption exchange between humidity and materials to spontaneously generate water adsorption gradients within the materials, thus achieving energy collection and efficient electrical conversion of ubiquitous environmental humidity.^8–10^ However, the humidity in the natural environment greatly fluctuates according to the factors such as temperature, climate, and geographical location, leading to intermittent power output. Therefore, it is necessary to develop humidity power generation technology that can produce high electrical output under extreme humidity, thus achieving a supply of long-term, remote, continuous power.^11,12^

Yao *et al.* utilized the confined nanopore structure to control the moisture absorption capacity of the material, avoiding the decrease or even disappearance of water adsorption gradients within the material by excessive moisture absorption in high humidity.^13,14^ Han *et al.* utilized high-density water-adsorption functional groups to achieve efficient collection of low humidity.^15,16^ The continuous advancement of design is necessary to develop reasonable materials to meet various humidity conditions, solve the problem of intermittent power output, and achieve continuous power generation in fluctuating environmental humidity.^17,18^

Polyoxometalates (POMs) contain numerous oxygen-containing functional groups and an inherent nanomorphology that align well with the design requirements of continuous power generation from fluctuating humidity.^19–23^ Their utility manifests in five key aspects: (i) the oxygen-rich surfaces readily form abundant hydrogen-bonding networks, ensuring efficient water collection from environmental humidity.^24–27^ (ii) The nanoscale dimensions and abundant nanopores of POMs enhance the efficiency of interaction with environmental humidity and limit excessive hygroscopicity, thus maintaining water adsorption gradients in fluctuating environmental humidity.^28–32^ (iii) Their semiconductor-like property and high surface charge density facilitate energy conversion for improved power generation performance.^33–35^ (iv) POMs act as electronic sponges that can reversibly accept and donate multiple electrons, which are expected to enable real-time feedback to external physical and chemical environments by charge-transfer under the interaction of external environments.^36–39^ (v) Their accurate structures and compositions, and structural and thermodynamic stability ensure long-term operational reliability in environmental humidity.^40–42^

**Scheme 1:**
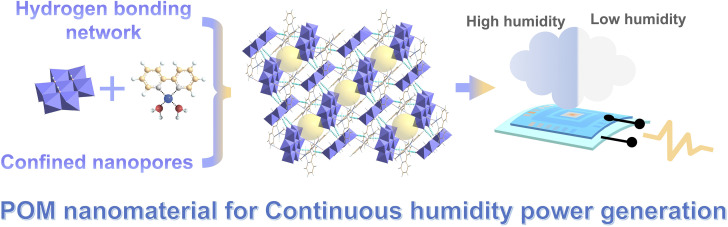
The continuous humidity power generation strategy proposed in this work. The POM is designed to efficiently collect environmental humidity with the abundant hydrogen bonds formed by oxygen-containing functional groups, and to maintain a water adsorption gradient with the confined nanopores. The POM device achieves continuous power generation in environments where there is fluctuating humidity.

Herein, the POM [Cu^II^(2,2′-bipy)(H_2_O)_2_Cl]_*n*_[Cu^II^-(2,2′-bipy)(H_2_O)_2_Al(OH)_6_Mo_6_O_18_]_*n*_ (Cu-CuAlMo_6_) with abundant oxygen-containing functional groups and confined nanopores was incorporated into a continuous humidity power generation strategy and tested (Scheme 1). Thin films of Cu-CuAlMo_6_ were formed and then assembled into devices that achieved continuous power generation under fluctuating environmental humidity (10–100%). The Cu-CuAlMo_6_ device spontaneously generated a voltage of 0.203 V and a current density of 4 µA cm^−2^ with a maximum power density of 0.06 µW cm^−2^ in 10% humidity. In 100% humidity with condensed water, the device maintained a voltage of 0.246 V and a current density of 14.5 µA cm^−2^, with a maximum power density of 0.214 µW cm^−2^ for 8 days. This device maintained its stability under mechanical stress (bending radius of 290 µm) and 50 operational cycles.

First-principles calculations, surface performance characterization, and electrochemical tests collectively revealed that the POMs efficiently collected humidity with the hydrogen bond networks formed by the abundant oxygen-containing groups. The humidity gradient was maintained by relying on the stable confined nanopores to achieve continuous electrical output. In addition, it generated a detectable electrical response within 0.1 s, and machine learning was used to establish a mathematical correlation between environments and electrical signals, thus achieving real-time multi-component sweat analysis by this single non-integrated POM device in a remote unsupervised state.

## Experimental

### Synthesis of the POMs

The POM [Cu^II^(2,2′-bipy)(H_2_O)_2_Cl]_*n*_[Cu^II^-(2,2′-bipy)(H_2_O)_2_Al(OH)_6_Mo_6_O_18_]_*n*_ was synthesized according to a previously reported method.^43^ CH_3_COOH (10 mL) was added to an aqueous solution (100 mL) of Na_2_MoO_4_·2H_2_O (3.5 g, 14.46 mmol). Next, 2,2′-bipyridine (0.2 g, 1.28 mmol) was added to a solution of water (15 mL) and methanol (25 mL). The two obtained solutions and Cu(NO_3_)_2_·2H_2_O (0.5 g, 2.06 mmol) were sequentially added to an aqueous solution (50 mL) of AlCl_3_·6H_2_O (1.5 g, 6.21 mmol). The pH of the mixed solution was adjusted to 2.6 using concentrated HCl at room temperature. Blue block-shaped crystals appeared within a week, and were then washed with water and dried at room temperature.

The POM Na_3_(H_2_O)_6_[Al(OH)_6_Mo_6_O_18_]·2H_2_O was synthesized according to a previously reported method.^44^ AlCl_3_·6H_2_O (1.5 g, 6.21 mmol) was added to a solution of water (25 mL) and CH_3_COOH (10 mL). Then, Na_2_MoO_4_·2H_2_O (3.5 g, 14.46 mmol) was added to the solution under vigorous stirring. The pH of the mixed solution was adjusted to 1.8 using concentrated HCl at room temperature. White crystals were obtained in a week, which were then washed with water and dried at room temperature.

### Preparation of POM devices

A flexible transparent indium tin oxide-polyethylene terephthalate (ITO-PET) conductive film was cleaned and placed in a mold. Cu-CuAlMo_6_ powder was ground for five minutes and dispersed in the mixed solvent of methanol and water (3 mg mL^−1^), with a volume ratio of methanol to water of 5 : 3. The liquid was ultrasonically treated for five minutes to prepare a uniform dispersion solution. The Cu-CuAlMo_6_ film was prepared by evaporating the solvent upon liquid-phase deposition in the mold. The Cu-CuAlMo_6_ film was aligned with printing holes for screen-printing. A conductive carbon electrode was obtained by applying and drying printing paste on the Cu-CuAlMo_6_ film to prepare the Cu-CuAlMo_6_ device.

### Test of electrical performance

All tests were completed using an electrochemical workstation CHI760e (Shanghai-Chenhua) and a digital source-meter Keithley 6517. Illumination was generated by a xenon lamp with an optical power density of 100 mW cm^−2^. The artificial sweat was prepared with NaCl (40 mmol L^−1^), KCl (9 mmol L^−1^), CaCl_2_ (5 mmol L^−1^), MgCl_2_ (0.6 mmol L^−1^), Na_2_HPO_4_ (10 mmol L^−1^), lactic acid (13.5 mmol L^−1^), urea (23.1 mmol L^−1^), and ascorbic acid (0.43 mmol L^−1^).

## Results and discussion

### The structure and charge transfer property of Cu-CuAlMo_6_ nanomaterials

**Fig. 1:**
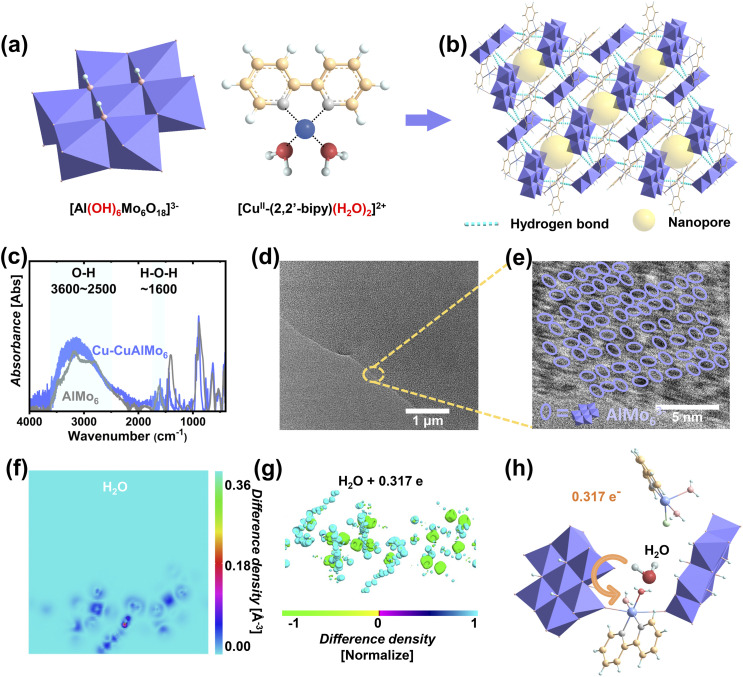
Structural and electronic characteristics of Cu-CuAlMo_6_ nanomaterials. (a) Atomic architecture of Cu-CuAlMo_6_, showing the polyoxoanion [AlMo_6_]^3−^ (blue polyhedrons), and ligands and cations (colored lines and spheres). (b) Highlighting the hydrogen-bonding network (cyan dashed lines) and confined nanopores (yellow spheres). (c) FTIR comparison of Cu-CuAlMo_6_ and AlMo_6_. (d) TEM of Cu-CuAlMo_6_. (e) High-resolution TEM of Cu-CuAlMo_6_ with AlMo_6_^3−^ circled in blue ellipses. (f) Two-dimensional projection of the differential charge density for Cu-CuAlMo_6_ with adsorbed H_2_O (color scale: electron density difference). (g) Three-dimensional differential charge density (color scale: normalized electron density difference). (h) Directional charge transfer, with orange arrows denoting the electron flow between Cu-CuAlMo_6_ and H_2_O.

According to the design requirements of continuous humidity power generation materials, Anderson-type polyoxoanions [Al(OH)_6_Mo_6_O_18_]^3−^ (AlMo_6_^3−^) with abundant hydroxyl groups (–OH) and [Cu^II^-(2,2′-bipy)(H_2_O)_2_]^2+^ ligands with coordination water groups were selected as building blocks (Fig. 1a). Hydroxyl groups and coordinating water, as oxygen-containing functional groups, can form hydrogen bonds and exhibit excellent hydrophilicity and hygroscopicity. The POM [Cu^II^(2,2′-bipy)(H_2_O)_2_Cl]_*n*_[Cu^II^-(2,2′-bipy)(H_2_O)_2_Al(OH)_6_Mo_6_O_18_]_*n*_ (Cu-CuAlMo_6_) was synthesized by a literature method, and the structural integrity was confirmed by Fourier transform infrared (FTIR) spectroscopy (Fig. S1a), powder X-ray diffraction (PXRD, Fig. S1b), and ion-mobility spectrometry-mass spectrometry (IMS/MS, Fig. S1c, with characteristic peaks in Table S1).^43^

The Anderson-type POM Na_3_(H_2_O)_6_[Al(OH)_6_Mo_6_O_18_]·2H_2_O (AlMo_6_) was synthesized and characterized (FTIR, XRD, and IMS/MS in Fig. S2 and Table S2).^44^ The identical spectral features of AlMo_6_^3−^ observed in both compounds proved the preservation of the polyoxoanion structure during the synthesis of Cu-CuAlMo_6_. Additional synthetic details are provided in the Experimental section.

The structural architecture of Cu-CuAlMo_6_ consists of anionic chains [Cu^II^-(2,2′-bipy)(H_2_O)_2_Al(OH)_6_Mo_6_O_18_]_*n*_^*n*−^ and cations [Cu^II^(2,2′-bipy)(H_2_O)_2_Cl]^+^, where the anionic chains are formed by the connection of AlMo_6_^3−^ (blue polyhedrons in Fig. S3) with bridging units {Cu^II^(2,2′-bipy)(H_2_O)_2_}^2+^. This structure was stabilized by hydrogen bonds (light blue dotted lines in Fig. 1b), as evidenced by FTIR spectroscopy (Fig. 1c). The broad absorption (3600–2500 cm^−1^ in Fig. 1c) was attributed to the O–H stretching vibration from terminal hydroxyl groups (–OH) and coordinated H_2_O. The enhanced intensity from Cu-CuAlMo_6_ (blue in Fig. 1c) to AlMo_6_ (gray in Fig. 1c) indicated additional hydrogen bonds.^45^ The redshifted H–O–H bending vibration (approximately 1600 cm^−1^ in Fig. 1c) was attributed to the decrease in bond energy and the increase in hydrogen bonds.

Transmission electron microscopy and energy dispersion X-ray analysis (TEM-EDX) demonstrated the uniformly distributed structure and elements of Cu-CuAlMo_6_ (Fig. 1d and S4). High-resolution TEM (HRTEM, Fig. 1e) displays the ordered arrangement of AlMo_6_^3−^, as shown in the dark area circled by the purple ellipses in the figure, which creates well-defined nanopores (orange spheres in Fig. 1b).

The adsorption and charge transfer characteristics of water molecules (H_2_O) on POM nanomaterials were investigated through molecular dynamics simulations (MDS) and first-principles calculations (Fig. 1f–h and S5).^46,47^ Energy minimization analysis revealed the preferential adsorption sites and binding configurations in the nanopores (Fig. S5a and S5b), which occupy the interior of the nanopores at sites on coordinated H_2_O of [Cu^II^-(2,2′-bipy)(H_2_O)_2_]^2+^ (Fig. S5c).

Two-dimensional differential charge density projections revealed different spatial charge distribution patterns: local charge transfer near adsorption sites (red/blue surfaces), electron recombination based on the molecular orbital structure of Cu-CuAlMo_6_, and charge delocalization involving the solution phase by hydrogen bonding structures (Fig. 1f). The three-dimensional differential charge density (Fig. 1g) showed a more intuitive charge transfer situation, with normalized difference density. Bader topology analysis (Fig. 1g) indicated an increased charge density of adsorbed H_2_O, proving that electrons were transferred from H_2_O to Cu-CuAlMo_6_, with an amount of 0.317 e (Fig. 1h).

The hydrogen-bond-mediated framework facilitates the transmission of electrical and chemical signals while providing charge transfer sites. The size of the nanopores was less than 1 nm, which is comparable to the size of Anderson-type polyoxoanions. The above tests demonstrate the excellent ability of Cu-CuAlMo_6_ to absorb environmental humidity by hydrogen-bonding networks formed by oxygen-containing functional groups.

### The preparation and properties of Cu-CuAlMo_6_ film

**Fig. 2:**
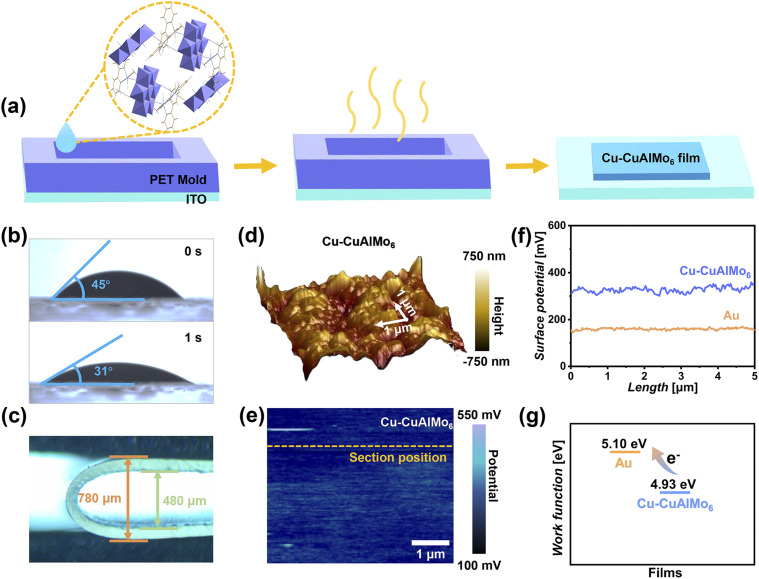
Fabrication and interfacial properties of Cu-CuAlMo_6_ films. (a) Schematic illustration of the solution-phase deposition process for film preparation. (b) Contact angle of water on the Cu-CuAlMo_6_ film (upper figure) and 1 s of contact (bottom figure). (c) Mechanical flexibility under bending stress. (d) Three-dimensional topographic profile showing the surface morphology (color scale: height). (e) Corresponding surface potential mapping (color scale: potential distribution) with the orange dashed line indicating the cross-sectional analysis position. (f) Comparison of the surface potential for Cu-CuAlMo_6_ (blue) with Au as a reference (orange). (g) Work function comparison between Au (5.10 eV) and Cu-CuAlMo_6_ (4.93 eV) film.

The Cu-CuAlMo_6_ film was fabricated *via* a solution-phase deposition method (Fig. 2a). A homogeneous dispersion was prepared by suspending Cu-CuAlMo_6_ powder in a mixed methanol/water solvent, which was then deposited onto flexible transparent ITO substrates through controlled solvent evaporation. XRD analysis confirmed that the structural integrity of Cu-CuAlMo_6_ was maintained during film formation (Fig. S6). The instantaneous surface water contact angle of 45° and the angle of 23° within 1 s demonstrate the extremely high surface hydrophilicity of Cu-CuAlMo_6_ films (Fig. 2b), which can effectively collect water molecules in ambient humidity.

The satisfactory UV-visible absorption in the ultraviolet region is shown in Fig. S7. The flexibility of the Cu-CuAlMo_6_ film was satisfactory, and it remained stable as it was bent into a U-shape at 180°, with a bending radius as small as 240 µm, and it fully recovered after pressure release (Fig. 2c). Kelvin probe force microscopy (KPFM) of a piece of 5 µm × 5 µm Cu-CuAlMo_6_ film revealed excellent surface uniformity, with a maximum surface fluctuation limited to <1 µm (Fig. 2d). Because of this combination of optical absorption, mechanical flexibility, and surface homogeneity, the Cu-CuAlMo_6_ films are particularly suitable for flexible device applications.

KPFM characterization revealed a uniform surface potential distribution (Fig. 2e), demonstrating the excellent uniformity in surface thickness and internal structure of the Cu-CuAlMo_6_ film, without aggregation.^48^ The Cu-CuAlMo_6_ film exhibited a high positive potential, with a surface potential of up to 320 mV, which was higher than that of 155 mV for Au (Fig. 2f and S8) and indicated the high surface negative charge density of the material. The surface potential results showed that the work function of the film is 4.93 eV, which is much lower than the 5.10 eV of Au (Fig. 2g). This proves the excellent electron-donating ability of Cu-CuAlMo_6_ films, which are expected to achieve efficient energy conversion and high electrical output.

### The structure and continuous humidity power generation performance of the Cu-CuAlMo_6_-based device

**Fig. 3:**
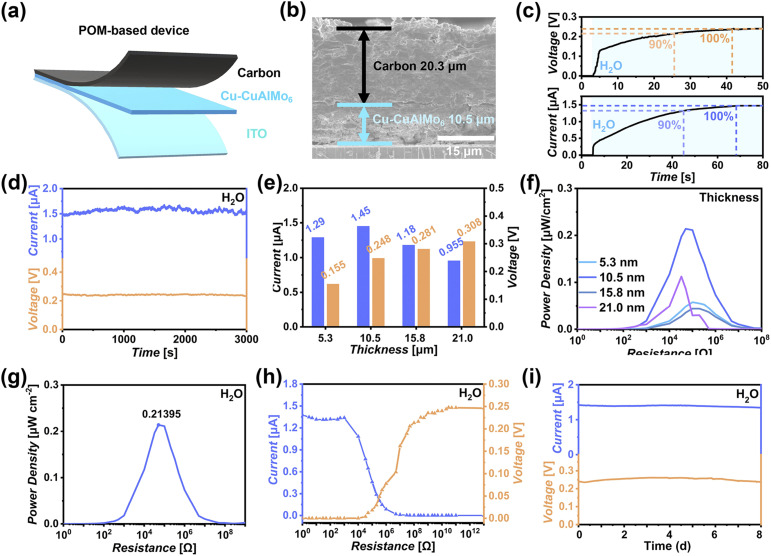
Structure and humidity power generation performance of the Cu-CuAlMo_6_-based device in high humidity with condensed water. (a) Schematic cross-section illustrating the multilayer device configuration. (b) Scanning electron micrograph revealing the well-defined interfacial layers in the assembled device. (c) Time-dependent current (top) and voltage (bottom) profiles generated in high humidity with condensed water. (d) Stable current and voltage outputs (blue: current; orange: voltage). (e) Thickness-related current and voltage outputs. (f) Thickness-related power density characteristics. (g) Highest power density generated at the optimal thickness. (h) Current and voltage output load resistances at the optimal thickness. (i) The stability of the Cu-CuAlMo_6_-based device under continuous operation in high humidity with condensed water.

The Cu-CuAlMo_6_-based device was fabricated through a scalable manufacturing approach combining liquid-phase deposition and screen-printing techniques, which is conducive to large-scale patterned manufacturing. The Cu-CuAlMo_6_ device consists of three layers (Fig. 3a): the screen-printed conductive carbon film for charge transfer (black), the Cu-CuAlMo_6_ active layer (blue), and the ITO counter electrode (cyan). Cross-sectional scanning electron microscopy (SEM) shows the structural integrity of the Cu-CuAlMo_6_-based device (Fig. 3b) with a uniform thickness and a clear interface.

The effective electrical output of the Cu-CuAlMo_6_-based device in high humidity is the most important parameter for evaluating the continuity and stability that is necessary for achieving continuous humidity power generation. The generation capability of the Cu-CuAlMo_6_-based devices with an effective area of 0.1 cm^2^ was characterized using an electrochemical workstation. Upon high environmental humidity, the open circuit voltage (*V*_OC_) and short circuit current (*I*_SC_) exhibited a rapid initial response, followed by a gradual increase before stabilizing at their maximum values (Fig. 3c). Key temporal parameters were quantified, including time to reach 90% of maximum *I*_SC_ and *V*_OC_ (*t*_90% *I*_sc__ and *t*_90% *V*_oc__) and time to achieve full stabilization (*t*_*I*_sc__ and *t*_*V*_oc__). The observed temporal asynchrony in the generation of electrical signals was attributed to the charge transfer and hydrogen-bond-mediated conduction processes of Cu-CuAlMo_6_.^49^

The device demonstrated stable humidity power generation performance, generating continuous *V*_OC_ and *I*_SC_ electrical signals under the environment of 100% humidity with condensed water (Fig. 3d). Thickness-dependent optimization studies revealed tunable *V*_OC_ and *I*_SC_ (Fig. 3e, f, S9 and Table S3), with maximum power density (0.21 µW cm^−2^, Fig. 3g) for 10.5 µm-thick films at a 51 K Ω load resistance (Fig. 3h). The highest power generation performance for the device was at approximately 40 °C (Fig. S10).

Long-term stability tests demonstrated that continuous operation occurred over 8 days without any significant signal attenuation (Fig. 3i), which proved the sustained water-activated self-power generation performance of the Cu-CuAlMo_6_-based device. The excellent continuous power generation capability under high humidity, even in the presence of condensed water, solves the problem of decreased or interrupted electrical output in humidity-driven power generation devices under such conditions.

**Fig. 4:**
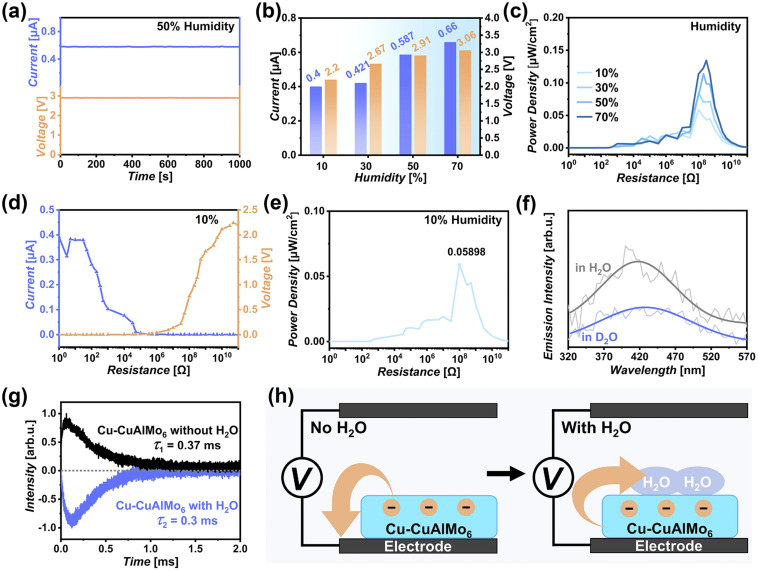
Humidity power generation performance and electricity generation mechanism of the Cu-CuAlMo_6_ device. (a) Stable current and voltage outputs of the Cu-CuAlMo_6_ device in 50% humidity (blue: current; orange: voltage). (b) Humidity-related current and voltage outputs. (c) Humidity-related power density characteristics. (d) Current and voltage output load resistance in 10% humidity. (e) Power density characteristics in 10% humidity. (f) Comparison of photoluminescence spectroscopy in H_2_O and D_2_O environments, demonstrating the hydrogen-bond mediated charge transfer. (g) Transient photoinduced voltage decay kinetics under anhydrous (black) and aqueous (blue) conditions with different charge decay. (h) Proposed electron transfer mechanisms under (left) anhydrous and (right) aqueous conditions, illustrating the water-mediated charge transport pathway.

Maintaining efficient electrical output under low humidity is an important step for achieving continuous electrical output in fluctuating natural environmental humidity. The Cu-CuAlMo_6_ devices showed stable power generation capacity under an average environmental humidity of 50%, and generated a stable *V*_OC_ and *I*_SC_ outputs of 2.91 V and 5.87 µA cm^−2^ (Fig. 4a), respectively, with a power density of 0.1139 µW cm^−2^ (Fig. 4b, c and Table S4). The device exhibited higher voltage in low humidity due to the more pronounced water adsorption gradient. By reducing the humidity to 10%, the devices displayed electrical signals of 2.2 V and 4 µA cm^−2^, with a power density of 0.059 µW cm^−2^ (Fig. 4d and e). During this change, the humidity decreased to 80%, but the performance only decreased to 48%, proving that there is excellent compatibility of the device with low environmental humidity, which was achieved by the efficient water capture ability of the hydrogen bonds in Cu-CuAlMo_6_.

The continuous output under the extreme humidity of 100% with condensed water and the efficient power generation under extremely low 10% humidity prove that the Cu-CuAlMo_6_ device can maintain continuous electrical output, as well as continuous power generation in fluctuating natural environments. This continuous power generation performance solves the problem of intermittent power generation under fluctuating humidity, and can provide continuous electrical energy for portable or remote devices without being limited by environmental fluctuations.

The charge-rich surface of Cu-CuAlMo_6_ films can induce an electrical double layer (EDL) at the Cu-CuAlMo_6_-humidity interface (Fig. S11), according to the KPFM results (Fig. 2e). The hydrogen bonds facilitate the efficient interfacial contact between the Cu-CuAlMo_6_ film and humidity, improving the charge transfer and power generation efficiency. Photoluminescence (PL) spectroscopy showed a pronounced decrease in emission intensity as the device environment was switched from H_2_O (gray in Fig. 4f) to D_2_O (blue in Fig. 4f), which was attributed to the stronger hydrogen bond interaction of D_2_O that promoted electron transfer and resulted in fluorescence quenching.^50^ The H/D exchange confirmed the existence of dynamic adsorption–desorption processes between Cu-CuAlMo_6_ and environmental humidity, and the proton/charge transfer occurred by the dynamic breaking and recombination of hydrogen bonds.^51^

Transient photoinduced voltage (TPV) measurements provide further mechanistic insights into the autonomous response process of the Cu-CuAlMo_6_ device. The TPV curve (black line in Fig. 4g) of the film tested under anhydrous conditions shows a decay time (*τ*) of 0.37 ms. The charge decay time was reduced to 0.3 ms by mixing a small amount of water into the testing system, which indicated that the presence of water accelerates the interface charge transfer performance (blue line in Fig. 4g). Notably, the TPV curve of the Cu-CuAlMo_6_ film shows that the signal reversed from upward to downward by adding water (Fig. 4g), which is caused by the charge reversal, and proved that the direction of electron transfer from Cu-CuAlMo_6_ to electrode changed to the direction from Cu-CuAlMo_6_ to water (Fig. 4h).^52^

In the left panel of Fig. 4h, the response process is shown between the Cu-CuAlMo_6_ film and electrode under an anhydrous environment, where the black rectangles represent electrodes, the blue rectangles represent Cu-CuAlMo_6_ films, and the orange spheres and arrows show the electrons and the direction of electron transfer, respectively. The electrons are quickly transferred from the Cu-CuAlMo_6_ film to the electrode in the absence of water, which is affected by the difference between the Cu-CuAlMo_6_ film and the electrode in surface charge density and work function. The electrons can rapidly transfer from the Cu-CuAlMo_6_ film to water as water is added (right panel of Fig. 4h), which mainly occurs due to the EDL and hydrogen bonding conduction.

The device utilizes environmental humidity to generate long-term stable DC output, proving that this Cu-CuAlMo_6_ humidity power generation process relies on the dynamic adsorption–desorption between Cu-CuAlMo_6_ and environmental humidity, thereby achieving continuous and stable energy conversion. The continuous environmental humidity power generation mechanism of the Cu-CuAlMo_6_ device dominated by hydrogen bonding and nanopores is subsequently proposed (Fig. S12):^53,54^ the adsorption of environmental humidity and the formation of a water adsorption gradient; the generation of an EDL and charge gradient at the Cu-CuAlMo_6_/water interface; the interaction and the proton/charge transfer by a dynamic hydrogen-bond network; and the dynamic humidity adsorption–desorption and hydrogen bonding reorganization for continuous power generation *via* environmental humidity.

### The ionic recognition and self-powered multi-component real-time monitoring capability of the Cu-CuAlMo_6_ device

**Fig. 5:**
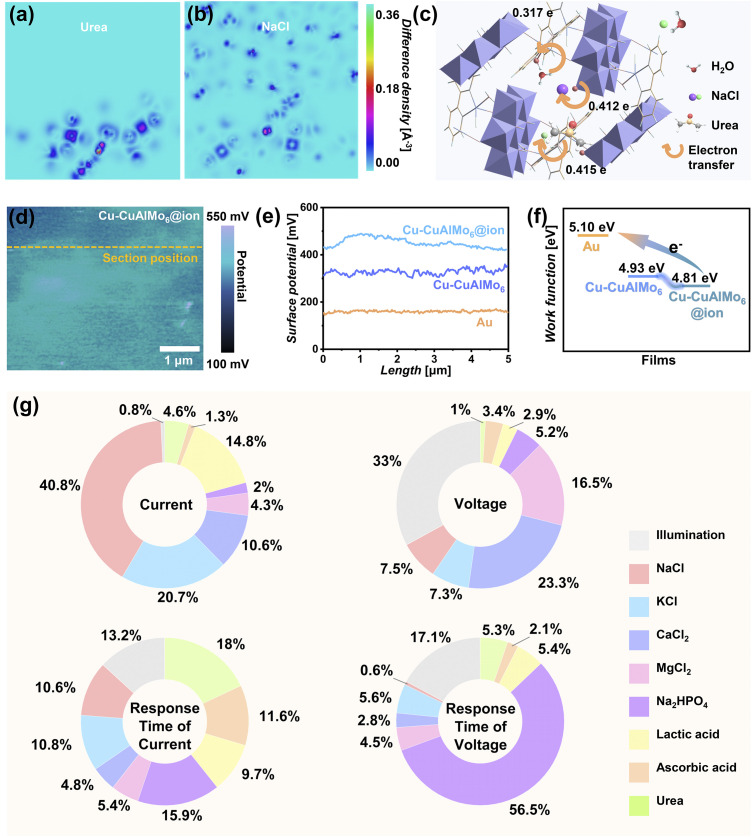
Ionic recognition and real-time monitoring of the Cu-CuAlMo_6_ device. (a and b) Differential charge density maps for Cu-CuAlMo_6_ adsorbed with (a) urea and (b) NaCl (Na^+^ and Cl^−^), illustrating ionic-specific charge redistribution (color scale: electron density difference). (c) Ionic adsorption sites and charge transfer in Cu-CuAlMo_6_. (d) Enhanced surface potential distribution with ion incorporation (orange dashed line: comparative analysis position, color scale: potential distribution). (e) Comparison of the surface potential for the Cu-CuAlMo_6_ film (blue), ion-incorporated film (cyan), and Au (orange). (f) Work function comparison between Au (5.10 eV), Cu-CuAlMo_6_ film (4.93 eV), and ion-incorporated Cu-CuAlMo_6_ film (4.81 eV). (g) Machine learning-derived feature analysis.

The excellent adsorption performance and charge transfer ability of Cu-CuAlMo_6_ may endow it with chemical recognition and monitoring functions, enabling real-time monitoring of environmental and chemical information while generating electricity under humidity. Molecular dynamics simulations (MDS) and first-principles calculations reveal different binding configurations of adsorbates in nanopores, such as urea and NaCl, as well as selective charge transfer (Fig. 5a, b and S13). Bader topology analysis indicates that Cu-CuAlMo_6_ adsorbs chemical substances and exhibits different charge transfer behaviors, and because of these characteristics, Cu-CuAlMo_6_ is expected to achieve chemical information recognition (Fig. 5c and S14). The FTIR and XRD results showed the excellent chemical stability of the Cu-CuAlMo_6_ nanomaterials (Fig. S15).

The Cu-CuAlMo_6_ film remained stable after introducing chemical components, with fluctuations less than 1 µm (Fig. S16a) and uniform charge distribution (Fig. 5d). Notably, the ionic interaction enhanced the surface potential from 320 mV to 410 mV (Fig. 5e and S16b), indicating the increased negative charge density (approximately 28% enhancement). The work function decreased from 4.93 eV to 4.81 eV (Fig. 5f), indicating an enhancement in the electron-donating capability. The X-ray photoelectron spectroscopy (XPS) analysis confirmed the charge transfer between the film and ionic components, which was manifested through the shifted characteristic binding energy (Fig. S17).^55–58^ This phenomenon likely arises from the synergistic effects of the POM's electronic structure, hydrogen-bond-mediated charge conduction, and ionic interactions within the nanopores.

The Cu-CuAlMo_6_ device demonstrated versatile recognition and analysis capabilities through its humidity-electrical signals influenced by environmental and chemical information. The light-modulated electrical signals (Fig. S18 and Table S5) enabled the real-time monitoring of illumination. For ionic analytes, *I*_SC_ and *V*_OC_ exhibited linear concentration dependence (Fig. S19a–S26a, orange for *V*_OC_ and blue for *I*_SC_), maintaining consistent response kinetics (*t*_*I*_sc__ and *t*_*V*_oc__, Fig. S19b–S26b). Distinct response patterns emerged between illumination and darkness (Fig.S19c–S26c, Table S6), providing dual-mode detection sensitivity.

Significantly varied electrical signatures were generated in solutions of identical concentration but different components (Fig. S27, Tables S7 and S8). An ionic recognition-feedback mechanism (Fig. S28) was revealed by electrochemical impedance spectroscopy (EIS, Fig. S29). As shown above, ions were adsorbed and recognized by POM from the selective charge transfer properties, changing the EDL and further affecting the transmission process of electrical signals to achieve feedback regulation of electrical signals (Fig. S28).

The mixed multi-component analysis capability of Cu-CuAlMo_6_ devices was systematically evaluated using artificial sweat and high-concentration mixed solutions (Fig. S30, S31, Tables S9 and S10).^59^ EIS characterization in artificial sweat revealed substantial differences in Nyquist and Bode results across compositions (Fig. S32), confirming that subtle compositional variations significantly impacted the interfacial charge transfer dynamics. The selective adsorption and charge transfer of the POMs (Fig. S33) exhibited specific interfacial charge behavior (Fig. S34), which generated modulated humidity electrical signals for real-time self-powered environmental and chemical information monitoring.

To establish robust mathematical models for multi-component analysis, the factors governing the humidity electrical signals of the Cu-CuAlMo_6_ device were systematically analyzed. Machine learning approaches were employed to decipher the complex relationships within the comprehensive dataset of component responses.^60^ Correlation analysis revealed key interdependencies among the electrical parameters, as visualized in the Pearson correlation heatmap (Fig. S35). The moderate linear correlation (|*r*| ≈ 0.4–0.6) between *I*_SC_ and *V*_OC_ reflects their coupled yet distinct response to environments, and the strong correlations (|*r*| > 0.7) between temporal parameters (*t*_*I*_sc__*vs. t*_90% *I*_sc__, and *t*_*V*_oc__*vs. t*_90% *V*_oc__) confirmed these response times as reliable metrics for quantifying the identification of solution components. These quantitative relationships provide the foundation for developing predictive models.

Machine learning models trained on the response relationships revealed distinct component-specific sensitivities in the Cu-CuAlMo_6_ device, as quantified by feature importance analysis (Fig. 5g). The *I*_SC_ exhibited predominant sensitivity to NaCl (40.8% contribution), demonstrating its exceptional responsiveness to ion variations, while the *V*_OC_ showed balanced modulation by illumination (33.0%) and CaCl_2_ (23.3%), reflecting competing photoactive and ionic-dependent charge transfer mechanisms.

Temporal response parameters displayed specialized analyte dependencies, with *t*_*I*_sc__ most influenced by urea (18.0%) and *t*_*V*_oc__ strongly governed by Na_2_HPO_4_ (56.5%). These quantitatively resolved sensitivity profiles established a complementary detection framework. The orthogonal coupling between specific electrical parameters and distinct analytes enabled deconvolution of complex multi-component systems through their unique electrochemical fingerprints, forming the basis for intelligent, self-powered platforms capable of multiplexed analysis.

**Fig. 6:**
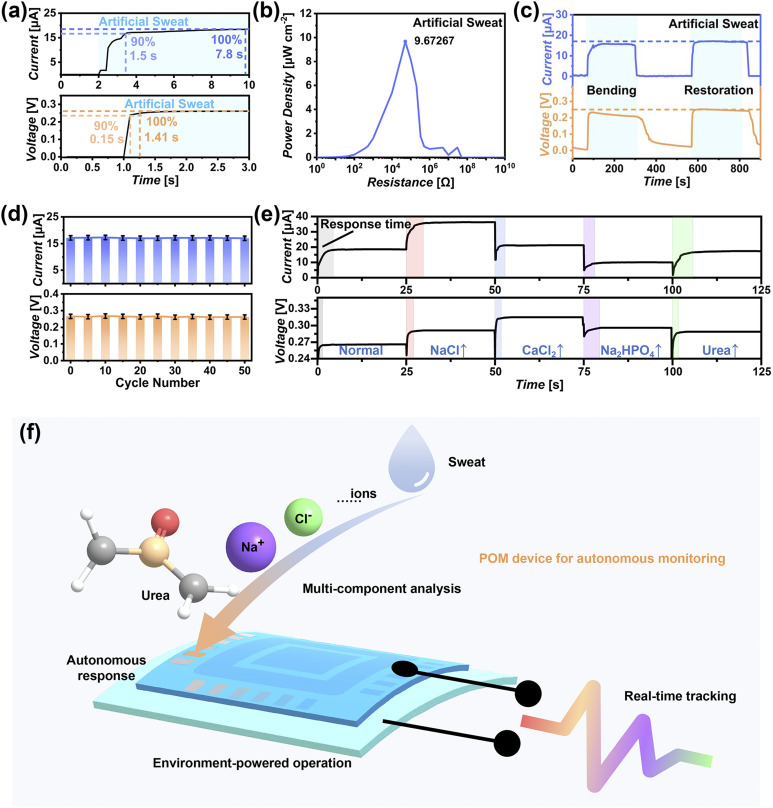
Actual operational performance of the Cu-CuAlMo_6_ device in self-powered real-time analysis. (a) Time-dependent current (top) and voltage (bottom) profiles generated in a sweat environment. (b) Maximum power density (10 µW cm^−2^ at 50 k Ω) as a function of load resistance. (c) Mechanical stability testing showing the maintained electrical output during bending (radius: 290 µm) and subsequent recovery. (d) Cyclic operational stability of the Cu-CuAlMo_6_ device. (e) Real-time multicomponent analysis *via* environment-responsive current/voltage signatures, with machine learning-enabled compositional decoding. (f) Operational schematic illustrating the autonomous monitoring strategy combining ionic feedback regulation with self-power generation.

The long-term continuous humidity power generation and multifunctional real-time monitoring application capability of the Cu-CuAlMo_6_ device in an actual natural environment were evaluated using artificial sweat as the monitoring sample. The Cu-CuAlMo_6_ device demonstrated rapid response kinetics in response to the artificial sweat (Fig. 6a), obtaining a stable *V*_OC_ (0.265 V) and *I*_SC_ (171 µA cm^−2^) within 1.4 s and 7.8 s, respectively (Fig. 6a and S34a). The self-generation power density of 10 µW cm^−2^ (Fig. 6b) was obtained at an optimal load resistance of 50 K Ω (Fig. S36b), representing a significant enhancement over the performance triggered by the high humidity (Fig. S36c).

This performance boost arose from the ionic feedback regulation. The components improve the electrical transmission performance, leading to an increase in *I*_SC_. The enhanced interfacial charge density at the POM/electrolyte increased the *V*_OC_. Remarkably, the device maintained outstanding operational stability, showing no degradation in *I*_SC_ or *V*_OC_ during the 14 day continuous testing in artificial sweat (Fig. S36b), which confirmed its long-term power generation performance. Additionally, it possesses excellent multi-component detection limits and analytical capabilities (Fig. S37).

The Cu-CuAlMo_6_ device exhibited exceptional mechanical flexibility, retaining 90% of its power generation efficiency and ionic recognition capability even under extreme bending conditions (180° bend, 290 µm radius; Fig. 6c and S38a). Complete performance recovery occurred upon stress release (Fig. 6c). The performance decreased to approximately 70% at smaller bending radii (<260 µm, Fig. S38b) due to electrode displacement. The flexibility of the device fully meets the requirements for daily wearables. Cycling tests confirmed the outstanding operational stability, with consistent *I*_SC_ (171 ± 2 µA cm^−2^) and *V*_OC_ (0.265 ± 0.004 V) maintained over 50 immersion cycles in artificial sweat (Fig. 6d), proving its reliability for repeated use.

The multi-component monitoring performance of the Cu-CuAlMo_6_ device was evaluated in artificial sweat with different components (Fig. 6e). The distinct response kinetics (*t*_*I*_sc__ and *t*_*V*_oc__) produced characteristic electrical fingerprints for each component (colored rectangular bars in Fig. 6e). The machine learning-assisted signal analysis accurately identified compositional changes in real-time, with results precisely matching actual variations in NaCl (red), CaCl_2_ (blue), Na_2_HPO_4_ (purple), and urea (green).

For the present POM device, multiple components were adsorbed by Cu-CuAlMo_6_, and electrons were selectively transferred, combining self-power generation with multi-component identification. Thus, real-time multi-component autonomous monitoring was achieved by a set of feedback-regulated electrical signals without additional power requirements (Fig. 6f). The device achieved biomimetic functions by the unique ionic feedback-regulation mechanism from component-specific charge transfer at the POM interface and hydrogen-bond-mediated signal transduction, completing the process of environmental response, feedback regulation, analysis, and expression in a manner similar to that of living organisms. Compared with traditional monitoring devices, this POM device exhibits a comprehensive performance of autonomous response, environment-powered self-operation, real-time perception, and multi-component analysis, and therefore, there are broad application prospects for its use.

## Conclusion

In this study, a new design strategy utilizing environmental humidity to achieve continuous power generation is proposed, which achieves a long-term continuous power supply under fluctuating environmental humidity. This continuous humidity generator is designed from POM nanomaterials with abundant oxygen-containing functional groups and confined nanopores, which form a self-sustaining water adsorption gradient in dynamic water exchange with humidity.

It demonstrates efficient and continuous hydroelectric performance and achieves three capabilities: (i) efficient electrical output in high humidity, even with condensed water, which is achieved by the stable water adsorption gradient maintained by confined nanoscale pores, and thus avoids gradient flattening by excessive moisture absorption. (ii) Efficient collection of H_2_O and power generation through low-humidity oxygen-containing hydrophilic groups and their hydrogen-bonding structure. (iii) Real-time self-powered monitoring and synchronous multicomponent detection through machine learning-assisted electrical fingerprint analysis in a single, non-integrated device that can remotely operate unsupervised.

This work demonstrates that precise regulation of the water adsorption process is crucial for improving device performance and promoting widespread application. The formation of the interface EDL structure also promotes the humidity-electric energy conversion process. Therefore, changing the work function of the device and increasing the surface charge density is expected to significantly improve the humidity power generation performance.

This work provides a reliable design strategy for developing continuous humidity power generation technology, solves the intermittent power supply problem of humidity generators in fluctuating humidity environments, and provides an important paradigm for achieving long-term continuous power supplies for remote unsupervised equipment. In addition, the real-time monitoring function in an external environment is a prerequisite for intelligent devices to autonomously adapt to environmental changes, adjust their actions, and complete fine and complex tasks safely and naturally. This work is expected to advance the development of intelligent next-generation devices to meet the challenges of humanization and autonomy.

## Author contributions

Tuo Ji: conceptualization, validation, investigation, writing – original draft. WeiLin Chen: methodology, resources, data curation, writing – review and editing, supervision, project administration, funding acquisition. Fan Liao: formal analysis, writing – review and editing. ZhenHui Kang: conceptualization, writing – review and editing, supervision, project administration, funding acquisition.

## Conflicts of interest

There are no conflicts to declare.

## Supplementary Material

SC-OLF-D6SC00287K-s001

## Data Availability

The data supporting this article have been included as part of the supplementary information (SI). Supplementary information: experimental conditions and materials, details of molecular dynamics simulations and first-principles calculations, results of characterizations and testing for materials and devices, and data analysis for electrochemical testing. See DOI: https://doi.org/10.1039/d6sc00287k.
