# Supplementary material for: Confined nanopores and hydrogen bonds of polyoxometalates for continuous electricity generation from fluctuating humidity
Source: Chem Sci. 2026 Apr 1;17(19):9468–80. doi: 10.1039/d6sc00287k (PMC13142679; doi:10.1039/d6sc00287k)
Supplement: SC-017-D6SC00287K-s001 [file SC-017-D6SC00287K-s001.pdf]

Supplementary Information (SI) for Chemical Science.
This journal is © The Royal Society of Chemistry 2026

Supplementary Information

Confined nanopores and hydrogen bonds of polyoxometalate for continuous electricity generation from fluctuating humidity

Tuo Ji [a], WeiLin Chen [a] *, Fan Liao [b] *, and ZhenHui Kang [b c] *

[a] *Key Laboratory of Polyoxometalate and Reticular Material Chemistry of Ministry of Education, Northeast Normal University, Changchun, 130024, P. R. China*

[b] *State Key Laboratory of Bioinspired Interfacial Materials Science, Institute of Functional Nano and Soft Materials (FUNSOM), Soochow University, Suzhou, 215123, P. R. China*

[c] *Macao Institute of Materials Science and Engineering (MIMSE), MUST-SUDA Joint Research Center for Advanced Functional Materials, Macau University of Science and Technology, Taipa, 999078, Macao, P. R. China*

*Corresponding author.

E-mail address: chenwl@nenu.edu.cn (WeiLin Chen); fliao@suda.edu.cn (Fan Liao); zhkang@suda.edu.cn and zhkang@must.edu.mo (ZhenHui Kang)

Supplementary Information

1. Experimental... 3

1.1 Materials 3

1.2 Characterizations 3

1.3 Calculation details 3

2. The structure and charge transfer property of Cu-$CuAlMo_6$ nanomaterials. 5

2.1 The characterization and structure of Cu-$CuAlMo_6$ material.. 5

2.2 The characterization and structure of $AlMo_6$ material. 7

2.3 The chemical structure of Cu-$CuAlMo_6$ framework. 9

2.4 The structure and charge transfer of Cu-$CuAlMo_6$ in water adsorption........................ 11

3. The properties of Cu-$CuAlMo_6$ film. 14

3.1 The basic properties of Cu-$CuAlMo_6$ film. 14

3.2 The charge transfer properties of Cu-$CuAlMo_6$ film.. 15

4. The continuous humidity power generation performance of the Cu-$CuAlMo_6$-based device. 16

4.1 The power generation performance of the Cu-$CuAlMo_6$ based device in high humidity of 100% with condensed water.. 16

4.2 The power generation performance of the Cu-$CuAlMo_6$ based device in low humidity. 18

4.3 The mechanism of Cu-$CuAlMo_6$ device for continuous humidity power generation.... 21

5 The ionic recognition and real-time monitoring performance of Cu-CuAlMo6 humidity power generation device. 25

5.1 The charge structure and charge transfer property of Cu-$CuAlMo_6$ nanomaterials in ionic adsorption. 25

5.2 The properties of Cu-$CuAlMo_6$ film with ions.. 29

5.3 The environmental illumination-response of the Cu-$CuAlMo_6$ device......................... 31

5.4 The ionic feedback-regulated electrical output of the Cu-$CuAlMo_6$ device. 33

5.5 The mechanism of Cu-$CuAlMo_6$ device for mixed multi-component monitoring. 47

5.6 The machine learning-assisted analysis of electrical signals generated by Cu-$CuAlMo_6$ devices. 49

6. The feasibility analysis of Cu-$CuAlMo_6$ device in practical application...................... 50

1. Experimental

1.1 Materials

All the raw materials used in the synthesis were purchased from the dealers and used directly without further purification.

1.2 Characterizations

The First-principles calculation were calculated on Vienna Ab initio Simulation Package (VASP). The powder X-ray diffraction (XRD) data were collected from a Bruker AXS D8 Advanced Bruker with Cu Kα radiation in the 2θ range from 5° to 80°. The Fourier transform infrared (FTIR) spectra were tested on AXS TENSOR-27 FTIR. Ion-Mobility Mass Spectrometry (IMS/MS) was performed ACQUITY UPLC H-Class PLUS/SYNAPT XS. Kelvin probe force microscope (KPFM) was performed using a Bruker AXS instrument under dust-free conditions. The X-ray photoelectron spectroscopy (XPS) was performed on a Thermo SCIENTIFIC ESCALAB 250Xi spectrometer by utilizing Al Kα radiation as the X-ray source. The transmission electron microscopy (TEM) was performed on a JEM-2100 PLUS. The field emission scanning electron microscopy (SEM) was characterized by HITACHI SU8010. The UV-Vis absorption was tested on cary60 UV-Visible Spectrophotometer the wavelength range of 200 nm-800 nm. The Photoluminescence Spectroscopy (PL) was tested on FLS1000.

1.3 Calculation details

The Forcite module on Materials Studio (MS) was used in Molecular Dynamics (MD) simulation, which is combined with COMPASS-III Field of Force for the optimization of adsorption site. This Field of Force covers the key terms of molecule interactions, including the

Van Der Waals Forces described by Lennard-Jones Potential Model and the chemical bond interactions fitted by Morse Potential Model. The Cut-off Radius of the Field of Force was set to 12 Å, and the Ewald Summation method was used to handle the long-range electrostatic interactions. The system was carried out under the Canonical Ensemble (NVT), and the temperature control was achieved by the Nose-Hoover chain method. The time step was set to 1 fs, and the total simulation time was 100 ps, ensuring a reasonable description of the molecular adsorption process. An explicit solvent model was chosen to consider the solvent effect of the system, and a periodic solvent box was constructed by TIP3P water molecules. The thickness of the vacuum layer in the unit cell is greater than 15 Å.

The calculation was performed by CASTEP, and the Generalized Gradient Approximation and Perdew-Burke-Ernzerhof (GGA-PBE) were employed to handle the electron exchange correlation effects. The charge distribution of adsorption was further clarified with Bader topology analysis. The DFT-D3 dispersion correction was used to accurately consider the weak interactions between molecules and surfaces. The ultra-soft pseudopotential (USPP) was selected to reduce the complexity of calculation. The cut-off energy of the plane-wave pseudo-potential was set to 500 eV to ensure the energy convergence and achieve the high accuracy. The selection of k-point mesh was based on the size of the unit cell and generated by the Monkhorst-Pack method. A 3 × 3 × 1 k-point mesh was used to ensure that the electronic properties of the surface structure are fully sampled. The self-consistent electronic convergence standard was set at $1*10^{-6}$ eV/atom to ensure the stable convergence of electron density. The convergence threshold of Atomic Force was set to 0.03 eV/Å in the process of geometric optimization to ensure the accuracy of structural optimization.

2. The structure and charge transfer property of Cu-CuAlMo6 nanomaterials.

2.1 The characterization and structure of Cu-CuAlMo6 material.

The FTIR result of the Cu-CuAlMo6 (blue in Fig. S1a) synthesized by the method reported in literature is consistent with the FTIR peak positions mentioned in the literature (orange in Fig. S1a), which are 1602 (m), 1473 (m), 1444 (m), 1317 (w), 1246 (w), 898 (s), 767 (m), 636 (s), 567 (w), 443 (m), where w represents the weak absorption peak, m represents the moderate absorption peak, and s represents the strong absorption peak. The measured XRD (blue in Fig. S1a) results are consistent with that reported in the literature (orange in Fig. S1b). The main peaks are marked with gray dotted lines. The IMS/MS result shows the material with different *m/z* of molecular fragments or clusters (Fig. S1c), and the specific formula of the molecular fragments or clusters corresponding to the main peaks marked by the orange triangle symbol are shown in the Table S1. The results of FTIR, XRD and IMS/MS effectively confirm the successful preparation and prove the structure of the Cu-CuAlMo6.

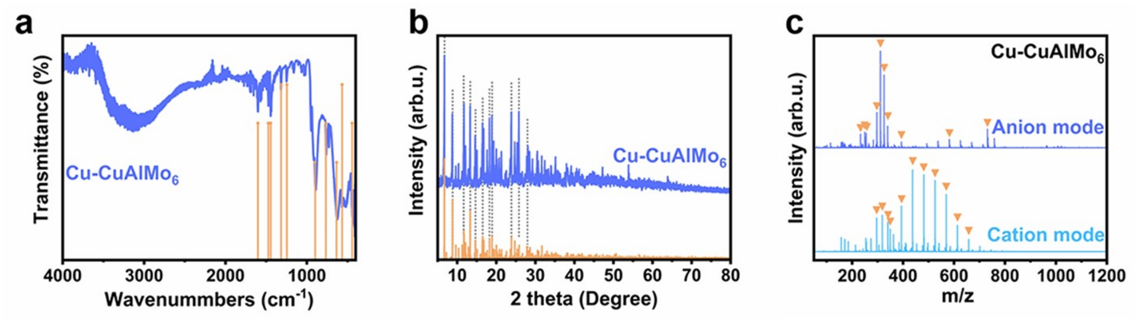

Fig. S1. Characterization of Cu-CuAlMo6 material.

a) The measured FTIR (blue) and the standard peak position reported in the literature (orange) of the Cu-CuAlMo6. b) The measured XRD (blue) and the standard peak position (orange) of the Cu-CuAlMo6. The main peak positions are marked with gray dotted lines. c) The IMS/MS

of the Cu-CuAlMo$_6$ material in anion (blue) and cation (cyan) mode. The important characteristic peaks are represented by triangular symbols.

Table S1. Characteristic IMS/MS peaks of Cu-CuAlMo$_6$.

m/z	Formula	Charge	mass
△233.15[a)]	$[Al(OH)_6Mo_6O_{18}](OH)_4(H_2O)_{32}$	-7	1637.17
249.15	$[Al(OH)_6Mo_6O_{18}]Cu(OH)_5$(2,2’-*b*ipy)$(H_2O)_{18}$	-6	1495.59
△255.23	$[Al(OH)_6Mo_6O_{18}](OH)_3(H_2O)_{27}$	-6	1530.09
297.15	$[Al(OH)_6Mo_6O_{18}](OH)(H_2O)_{10}$	-4	1189.81
△311.16	$[Al(OH)_6Mo_6O_{18}](OH)(H_2O)_{13}$	-4	1243.86
△325.19	$[Al(OH)_6Mo_6O_{18}](OH)(H_2O)_{16}$	-4	1297.91
△339.20	$[Al(OH)_6Mo_6O_{18}](OH)(H_2O)_{19}$	-4	1351.95
△439.25	$[Al(OH)_6Mo_6O_{18}](H_2O)_{18}$	-3	1316.93
581.30	$[Al(OH)_6Mo_6O_{18}]CuCl(H_2O)_4$	-2	1163.71
731.03	$[Al(OH)_6Mo_6O_{18}]_2Cu$(2,2’-*b*ipy)$(H_2O)_{40}$	-4	2925.65
296.15	[Cu-CuAlMo$_6$][Cu(2,2’-bipy)]$_5(H_2O)_{18}$	+10	2962.55
318.16	[Cu-CuAlMo$_6$][Cu(2,2’-*b*ipy)]$_6(OH)_2(H_2O)_{16}$	+10	3180.26
340.18	[Cu-CuAlMo$_6$][Cu(2,2’-*b*ipy)]$_7(OH)_4(H_2O)_{14}$	+10	3397.98
362.19	[Cu-CuAlMo$_6$][Cu(2,2’-*b*ipy)]$_8(OH)_6(H_2O)_{12}$	+10	3615.69
393.21	$[Al(OH)_6Mo_6O_{18}]$Cu[Cu(2,2-*b*ipy)]$_3(H_2O)_{14}$	+5	1967.60
437.23	$[Al(OH)_6Mo_6O_{18}]$Cu[Cu(2,2-*b*ipy)]$_4(OH)_2(H_2O)_{12}$	+5	2185.32
481.26	$[Al(OH)_6Mo_6O_{18}]$Cu[Cu(2,2-*b*ipy)]$_5(OH)_4(H_2O)_{10}$	+5	2403.03
525.29	$[Al(OH)_6Mo_6O_{18}]$Cu[Cu(2,2-*b*ipy)]$_6(OH)_6(H_2O)_8$	+5	2620.74
569.31	$[Al(OH)_6Mo_6O_{18}]$Cu[Cu(2,2-*b*ipy)]$_7(OH)_8(H_2O)_6$	+5	2838.46
613.34	$[Al(OH)_6Mo_6O_{18}]$Cu[Cu(2,2-*b*ipy)]$_8(OH)_{10}(H_2O)_4$	+5	3056.17
657.36	$[Al(OH)_6Mo_6O_{18}]$Cu[Cu(2,2-*b*ipy)]$_9(OH)_{12}(H_2O)_2$	+5	3273.88

[a)] The symbol “△” represents the peaks in both Cu-CuAlMo$_6$ and AlMo$_6$.

2.2 The characterization and structure of $AlMo_6$ material.

Anderson type POM $Na_3(H_2O)_6[AlMo_6O_{24}H_6]\cdot 2H_2O$ ($AlMo_6$, Fig. S2a, S2b, and S2c) with the same polyoxoanions as Cu-$CuAlMo_6$ is prepared by the method reported in the literature, which has the main characteristic peak positions of FTIR reported in the literature are 1620 (m), 947 (s), 920 (s), 845 (w), 650 (s), 574 (m), 530 (w), 447 (w). The FTIR shows a high degree of agreement between the Cu-$CuAlMo_6$ and $AlMo_6$, indicating that the basic structure of polyoxoanions is retained and unchanged in the Cu-$CuAlMo_6$ (Fig. S2d). The Cu-$CuAlMo_6$ (blue) and $AlMo_6$ (grey) are different in the XRD positions (Fig. S2e), indicating a significant difference in their crystallization properties. And the polyoxoanions does not maintain its original crystal morphology as the one-dimensional material Cu-$CuAlMo_6$ is formed.

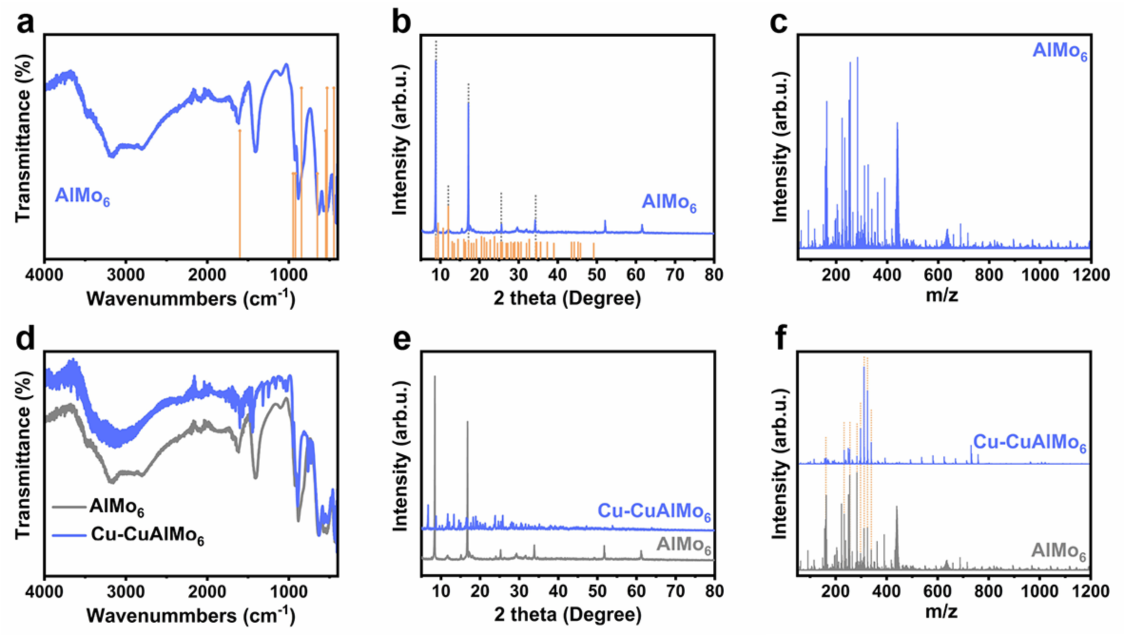

Fig. S2. Comparative analysis of Cu-$CuAlMo_6$ and $AlMo_6$.

a) The FTIR result (blue) and standard peak position (orange) of $AlMo_6$. b) The XRD result (blue) and standard peak position (orange) of $AlMo_6$. c) The IMS/MS result of the $AlMo_6$ material in anion mode. d) The comparison of FTIR results between Cu-$CuAlMo_6$ (blue) and $AlMo_6$ (grey). e) The comparison of XRD results between Cu-$CuAlMo_6$ (blue) and $AlMo_6$

(grey). f) The comparison of IMS/MS results between Cu-$CuAlMo_6$ (blue) and $AlMo_6$ (grey), where the consistent main characteristic peaks are marked with orange dotted lines.

Multiple identical *m/z* peaks of IMS/MS results are observed in both Cu-$CuAlMo_6$ and $AlMo_6$ materials, which are corresponding to the molecular fragments or clusters formed by the same Anderson type polyoxoanions (Fig. S2f). The molecular formulas to the main peaks are shown in the Table S2, demonstrating the retention of the polyoxoanion structure and the excellent stability of the Cu-$CuAlMo_6$.

Table S2. Characteristic IMS/MS peaks of $AlMo_6$.

m/z	Formula	Charge	mass
162.89	$[Al(OH)_6Mo_6O_{18}](OH)4(H_2O)_4$	-7	1132.74
△233.15	$[Al(OH)_6Mo_6O_{18}](OH)_4(H_2O)_{32}$	-7	1637.17
250.15	$[Al(OH)_6Mo_6O_{18}]_2(OH)_3(H_2O)_{12}$	-9	2252.51
△255.23	$[Al(OH)_6Mo_6O_{18}](OH)_3(H_2O)_{27}$	-6	1530.09
283.27	$[Al(OH)_6Mo_6O_{18}](OH)(H_2O)_7$	-4	1135.77
△311.16	$[Al(OH)_6Mo_6O_{18}](OH)(H_2O)_{13}$	-4	1243.86
△325.19	$[Al(OH)_6Mo_6O_{18}](OH)(H_2O)_{16}$	-4	1297.91
△339.21	$[Al(OH)_6Mo_6O_{18}](OH)(H_2O)_{19}$	-4	1351.95
△439.17	$[Al(OH)_6Mo_6O_{18}](H_2O)_{18}$	-3	1316.93

2.3 The chemical structure of Cu-CuAlMo$_6$ framework.

The chain like structural composition of Cu-CuAlMo$_6$ is shown in Fig. S3, where the Anderson type polyoxoanions are represented as the blue polyhedrons, and the atoms are represented by spherical shapes of various colors. In the Cu-CuAlMo$_6$, the Anderson type polyoxoanions $[Al(OH)_6Mo_6O_{18}]^{3-}$ ($AlMo_6^{3-}$, blue polyhedrons in Fig. S3) are connected with the transition metal complexes $[Cu^{II}(2,2'$-*b*ipy$)(H_2O)_2]^{2+}$ (the structure of the ball stick model with double rings in the Fig. S3) to form the chain like anion structure $[Cu^{II}$-(2,2'-*b*ipy)$(H_2O)_2Al(OH)_6Mo_6O_{18}]_n^{n-}$. And the copper chloride complexes $[Cu^{II}(2,2'$-*b*ipy$)(H_2O)_2Cl]^+$ are the cations (the structure with double rings and connected with green Cl). The alternating appearance with two configurations of Anderson type polyoxoanions result in a one-dimensional chain with helical structure.

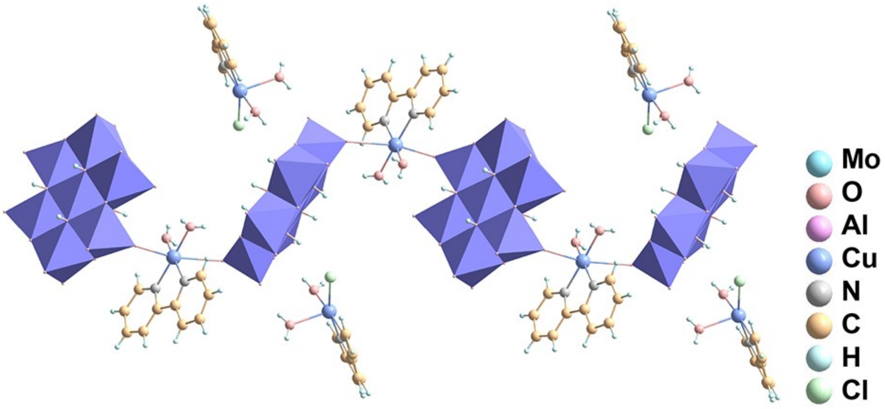

Fig. S3. The structure of Cu-CuAlMo$_6$ framework.

The spiral structure of the Cu-CuAlMo$_6$ material is determined by the hydrogen bonds intra the chain. The hydrogen bonds intra the chain are formed between the H_2O molecules coordinated with copper atoms in $[Cu^{II}(2,2'$-*b*ipy$)(H_2O)_2]^{2+}$ and two oxygen atoms on two Anderson anions connected with $[Cu^{II}(2,2'$-*b*ipy$)(H_2O)_2]^{2+}$ (one of the oxygen atoms is the

terminal oxygen atom of the Anderson polyoxoanions $AlMo_6^{3-}$ and the other one is the bridge oxygen atom of the other $AlMo_6^{3-}$). The spiral structure is also influenced by hydrogen bonding interactions inter chains. One is the hydrogen bond between the hydroxyl group (-OH) in the polyoxoanions $AlMo_6^{3-}$ and the terminal oxygen atom in the $AlMo_6^{3-}$ on adjacent chain, and the other is the hydrogen bond between the H_2O molecules coordinated with Cu in $[Cu^{II}$(2,2'-*b*ipy)$(H_2O)_2]^{2+}$ and the terminal oxygen atoms in the $AlMo_6^{3-}$ on adjacent chain. The inter chain and intra chain hydrogen bonds form the hydrogen bonding supramolecular network in the Cu-$CuAlMo_6$ material.

The TEM-EDX results of the Cu-$CuAlMo_6$ material show that the morphology of the material is uniform (Fig. S4), with elements of Cu (purple), Al (cyan), Mo (yellow), O (red), Cl (green), C (orange), and N (blue) evenly distributed in the material, indicating that the structure of the Cu-$CuAlMo_6$ material is stable.

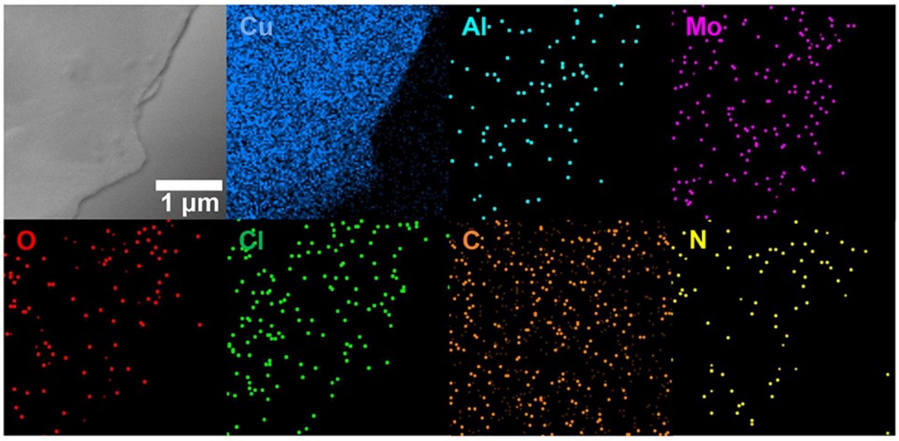

Fig. S4. EDX results of Cu-$CuAlMo_6$ material.

The EDX result of the Cu-$CuAlMo_6$ material with elements of Cu (purple), Al (cyan), Mo (yellow), O (red), Cl (green), C (orange), and N (blue).

2.4 The structure and charge transfer of Cu-$CuAlMo_6$ in water adsorption.

The periodic solvent box constructed by TIP-3P water molecules can ensure the effective influence of water molecules on the system. The vacuum layer thickness of the unit cell is greater than 15 Å, which avoids mirror interactions between the surfaces and improves the accuracy of adsorption.

The molecular dynamics simulation results show that H_2O molecules are effectively adsorbed in the Cu-$CuAlMo_6$, and the stable adsorption sites are close to the coordinated H_2O molecules and polyoxoanions in the Cu-$CuAlMo_6$ (Fig. S5a). The energy of the adsorption system is analyzed to further determine the adsorption sites of H_2O molecules on the Cu-$CuAlMo_6$. Fig. S5b shows the energy distribution of H_2O adsorbed by Cu-$CuAlMo_6$ along the C-axis direction, with the horizontal axis representing the normalized values along the C-axis direction and the vertical axis representing the change in ion number density per cubic Bohr radius volume. The adsorption is spontaneous as the value shown in Fig. S5b is negative. The lowest value indicates that the stable adsorption site between H_2O molecules and Cu-$CuAlMo_6$ is located on the H_2O molecules coordinated with copper in the anionic structure (Fig. S5c). The adsorbed substances and adsorption sites on the Cu-$CuAlMo_6$ are highlighted by atoms of O (red), H (white). It can be observed that the adsorption behavior of H_2O molecules by Cu-$CuAlMo_6$ mainly occurs within the nanopores (Fig. S5c).

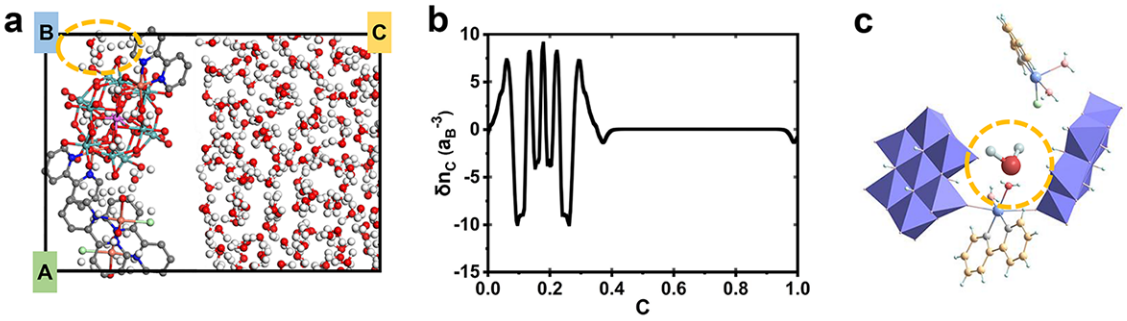

Fig. S5. The molecular dynamics simulation of Cu-$CuAlMo_6$ with adsorbate H_2O.

a) The molecular dynamics simulation results of H_2O (circled by an orange dotted line) adsorbed by Cu-$CuAlMo_6$. b) The adsorption energy of H_2O molecules adsorbed by Cu-$CuAlMo_6$ along the C-axis direction. c) The adsorption site of H_2O molecules adsorbed by Cu-$CuAlMo_6$ in aqueous solution.

Differential charge density analysis ($\Delta\rho=\rho_{system}$ - $\rho_{adsorbate}$ - $\rho_{material}$) is used to quantitatively characterize the interface charge transfer, which reveals the transfer and redistribution of electrons during the adsorption process by comparing the differences between the total charge density of the system, the adsorbate, and the material. The DFT-D3 dispersion correction is used to ensure the accurate description of interactions and optimize long-range interactions of molecular surface, which is crucial for the study of molecular adsorption. The electron ion interactions are more accurately described by ultra-soft pseudopotential with good accuracy for various material systems. The three-dimensional differential charge density of H_2O molecule adsorbed by the Cu-$CuAlMo_6$ shows the charge rearrangement (Fig. 1g) with normalized range of -1 - 1 $Å^{-3}$.

According to the two-dimensional projection of Differential charge density result (Fig. 1f), charge transfer occurs not only at the adsorption sites between the Cu-$CuAlMo_6$ and H_2O molecules. Cu-$CuAlMo_6$ has charge delocalization property from the unique molecular orbital structure, leading to electronic reconstruction within Cu-$CuAlMo_6$ molecules. Water molecules in aqueous solution have weak charge delocalization from hydrogen bonding effects. Therefore, charge transfer occurs at adsorption sites, in polyacid nanomaterials, and in solutions.

Two-dimensional projection of Differential charge density reveals different spatial charge distribution patterns: local charge transfer near adsorption sites (red/blue surfaces), electron recombination from the unique molecular orbital structure of Cu-$CuAlMo_6$, and charge delocalization involving the solution phase from hydrogen bonding structures in aqueous environments (Fig. 1f).

Bader topology analysis can accurately identify the charge rearrangement of adsorption sites, helping to clarify the changes in adsorption sites and their charge environments. The charges are significantly rearranged as the H_2O molecule is adsorbed by the Cu-CuAlMo$_6$. The charge of the H_2O molecule adsorbed by the Cu-CuAlMo$_6$ is 0.317e (Fig. 1g). The positive value indicates an increase in the charge density on the adsorbed H_2O molecule, that is, the electrons move out of the adsorbed H_2O.

3. The properties of Cu-$CuAlMo_6$ film.

3.1 The basic properties of Cu-$CuAlMo_6$ film.

The XRD results show that the XRD of the Cu-$CuAlMo_6$ film (blue in Fig. S6) is consistent with that of Cu-$CuAlMo_6$ material (gray in Fig. S6), which indicates that the basic properties of the Cu-$CuAlMo_6$ material is unchanged with preparation process of the Cu-$CuAlMo_6$ film.

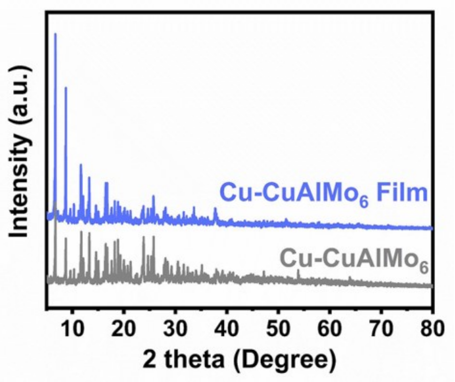

Fig. S6. XRD comparison of Cu-$CuAlMo_6$ film and powder.

The Cu-$CuAlMo_6$ film exhibits good light absorption in the ultraviolet and visible light regions of 600 nm-800 nm (Fig. S7).

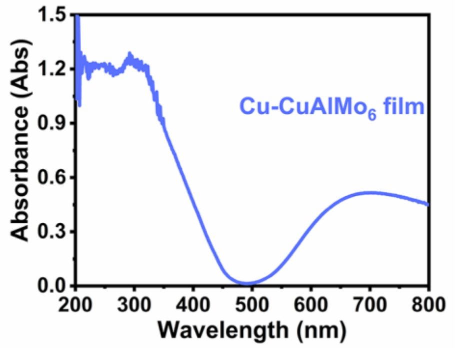

Fig. S7. UV-visible absorption spectrum of Cu-$CuAlMo_6$ film.

3.2 The charge transfer properties of Cu-$CuAlMo_6$ film.

In the KPFM results, the measured surface potential is actually the contact potential between the conductive probe of the atomic force microscope and the sample. In our results, the measured positive potential represents that the work function of the probe is higher than that of the Cu-$CuAlMo_6$ material, and the higher the potential, the greater the difference in work function, making it easier for the Cu-$CuAlMo_6$ material to donate electrons. Therefore, the distribution of surface potential difference in KPFM can reflect the electron donating ability of the Cu-$CuAlMo_6$ material surface, that is, the distribution state of surface negative charges. In addition to the properties of the Cu-$CuAlMo_6$ material itself, the thickness and uniformity of phase structure can also have a certain impact on the KPFM results.

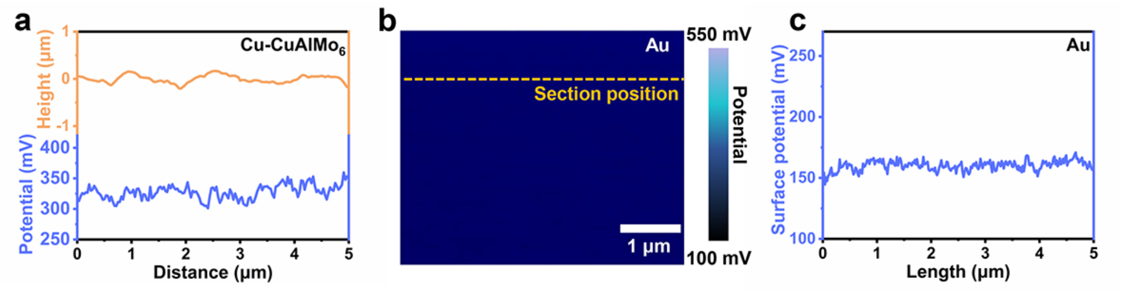

Fig. S8. KPFM surface potential analysis of Cu-$CuAlMo_6$ film and Au film.

a) The surface height (orange) and surface potential (blue) with the section position at the orange line on the Cu-$CuAlMo_6$ film in Fig. 2e. b) The two-dimensional surface potential distribution of the Au film. c) The surface potential (blue) with the section position at the orange line on the Au film in Fig. S8b.

4. The continuous humidity power generation performance of the Cu-CuAlMo6-based device.

4.1 The power generation performance of the Cu-CuAlMo6 based device in high humidity of 100% with condensed water.

The different triggered short circuit current (I_{SC}) and open circuit voltage (V_{OC}) are generated by the Cu-CuAlMo6 devices with different thicknesses of Cu-CuAlMo6 film, which are triggered by high humidity of 100% with condensed water and exhibited the highest output power performance at a thickness of 10.5 μm (Fig. S9 and Table S3).

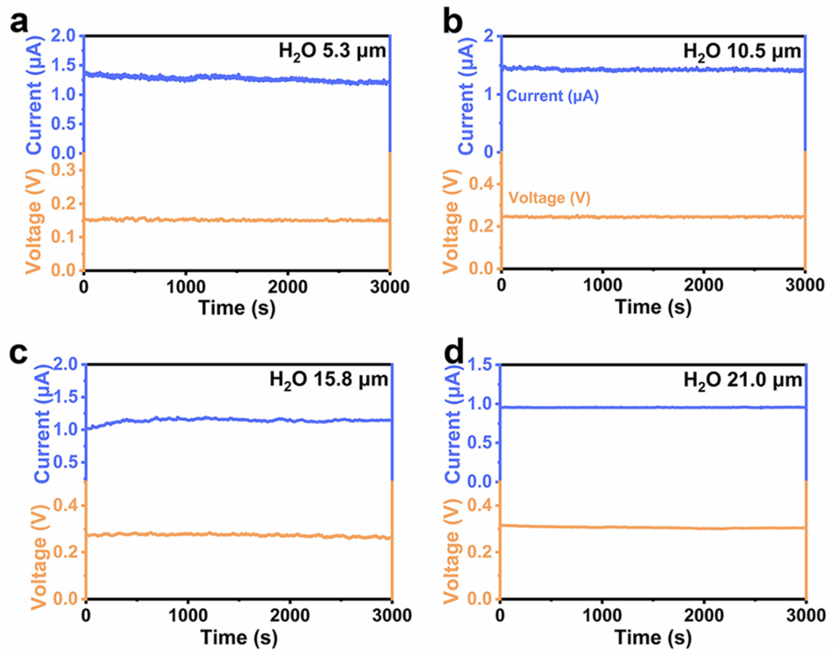

Fig. S9. Thickness-dependent environment response of Cu-CuAlMo6 devices.

a-d) The triggered electrical signals of the Cu-CuAlMo6 devices triggered by deionized water with the thicknesses of 5.3 μm (a), 10.5 μm (b), 15.8 μm (c), and 21.0 μm (d).

As the thickness increases, the I_{SC} of the Cu-CuAlMo$_6$ device shows a trend of firstly increasing and then decreasing, with the highest I_{SC} of 1.44 μA (with the area of the Cu-CuAlMo$_6$ device is 0.1 cm^2). The V_{OC} shows an upward trend as the thickness increases, and the changing trend slows down. The Cu-CuAlMo$_6$ device achieves the highest performance with the I_{SC} of 1.44 μA, the V_{OC} of 0.248 V, and the output efficiency of about 0.21 μW cm^{-2} at a thickness of 10.5 μm, where the thickness is expressed as the thickness of the Cu-CuAlMo$_6$ film.

Table S3. Thickness-dependent hydrovoltaic self-power generation performance of Cu-CuAlMo$_6$ devices triggered high humidity with condensed water.

Thickness [μm]	I_{SC} [μA]	V_{OC} [V]	Output power density [μW cm^{-2}]
5.3	12.9	0.155	0.058
10.5	1.48	0.248	0.214
15.8	1.18	0.281	0.113
21.0	0.955	0.308	0.044

4.2 The power generation performance of the Cu-$CuAlMo_6$ based device in low humidity.

The I_{SC} and V_{OC} of Cu-$CuAlMo_6$ devices are generated by the different humidity of 10% -70% with the thickness of 10.5 μm (Table S4). As the humidity increases, the I_{SC} and V_{OC} of the Cu-$CuAlMo_6$ devices show a trend of increasing. The devices at 10% -70% humidity generate higher V_{OC} and lower I_{SC} compared with the high humidity containing condensed water, which may be due to the significant water adsorption gradient and reduced hydrogen bond conduction at low humidity.

The generation of voltage originates from the charge exchange gradient caused by the water adsorption gradient on the inner and outer surfaces of the device, thereby forming a potential. Restricted nanopores can limit the sustained deep adsorption of water molecules. The higher the environmental humidity, the higher the water content on the outer surface of the material, and the deeper it goes into the interior of the film from the outer surface, the lower the water content until it reaches zero or touches the inner surface. Therefore, the voltage generated by the device is closely related to the environmental humidity and humidity absorption thickness. The water adsorption at high humidity of 100% is significantly higher than at low humidity, and the moisture content on the inner surface is higher, resulting in the decreased adsorption gradient and a lower voltage. Therefore, as shown in the Fig. 3e, the increase in thickness can lengthen the path of water adsorption, leading to an increase in the water adsorption gradient and causing an increase in voltage. At low humidity, the internal water molecule content approaches zero, and the water adsorption gradient reaches its limit, thus the device exhibits extremely high voltage (Fig. 4b and Table S4). The more efficient water exchange process, superior proton conductivity, and higher charge by high humidity adsorption can eliminate the decrease in power generation efficiency caused by the decrease in adsorption gradient, thereby achieving higher power generation efficiency.

Table S4. Humidity-dependent self-power generation performance of Cu-$CuAlMo_6$ devices.

Humidity [%]	I_{SC} [μA]	V_{OC} [V]	Output power density [μW cm^{-2}]
10	0.4	2.2	0.0590
30	0.421	2.67	0.0851
50	0.587	2.91	0.1139
70	0.66	3.06	0.1344

The representation of relative humidity values is temperature dependent, so the environments with lower temperatures have lower water content under the same relative humidity values. Therefore, take the humidity of 70% at 10 °C as the standard moisture content, maintaining the constant environmental moisture content and changing the temperature, and testing the humidity power generation performance of the Cu-$CuAlMo_6$ device (Fig. S10). The results show that the humidity power generation performance (voltage, current, and power density) of the Cu-$CuAlMo_6$ device are firstly increased and then decreased with the increase of temperature from 10 °C to 70 °C (Fig. S10a). And the structure and properties of the Cu-$CuAlMo_6$ nanomaterials remain stable under this temperature change. The device has the highest power generation performance at around 40 °C (Fig. S10b), which may stem from its temperature dependent adsorption capacity for environmental water molecules and the hydrogen bond charge conductivity within the device.

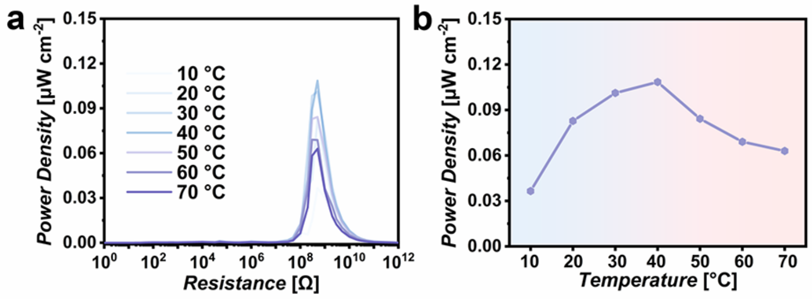

Fig. S10. Temperature stability of Cu-$CuAlMo_6$ devices.

a) The output power density of the device in environments with the same water content but different temperatures. b) The variation curve of device output power density with temperature.

4.3 The mechanism of Cu-CuAlMo$_6$ device for continuous humidity power generation.

The theoretical calculation results show the charge transfer during the water adsorption process, reflecting the charge transfer during the process of POM and water molecules from independent to stable adsorption. And water molecules transfer a certain number of electrons to POM molecules to form a steady state. The TPV results show the electrochemical charge transfer process, which is influenced by surface charge density and interfacial EDL. The EDL structure is formed as water comes into contact with the surface of POM with high surface negative charge density, resulting in a high potential from water to the device at this interface. Under the influence of this potential, electrons can transfer from the POM film to the water, which does not change the charge density of the POM and water molecules, but forms an effective channel for charge transfer, promoting rapid transfer of interfacial charges.

The EDL is represented by the yellow and gray circles in light gray aqueous solution (Fig. S11). The protons, electrons, and negatively charged ions are respectively represented by orange, blue and gray circles. The KPFM shows that the negative charges (blue circles) are distributed on the surface of Cu-CuAlMo$_6$ film. The positive charges in the water are firmly adsorbed by the surface of Cu-CuAlMo$_6$ film with high negative charges, forming a surface ion layer (stern layer, orange circles). The EDL structure is formed by the stern layer and the diffusion layer (gray circles) which is rich in counter ions on the surface of the Cu-CuAlMo$_6$ film. A strong electric field is formed in the EDL by the aggregated surface ions and counter ions, resulting in a large potential gradient.

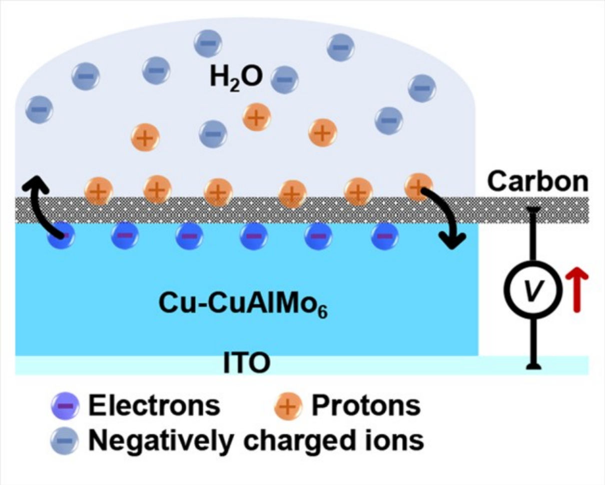

Fig. S11. EDL formation at POM-water interface.

Firstly, the surface of the Cu-CuAlMo$_6$ film adsorb environmental humidity and form water adsorption gradient (Fig. S12a). The H_2O molecules can interact with the hydrophilic terminals to form hydrogen bonds by the oxygen-containing active sites such as the terminal oxygen atoms, coordinated H_2O molecules and the hydroxyls in Cu-CuAlMo$_6$ (Fig. 1b). The hydrogen bond network of Cu-CuAlMo$_6$ can also act as active sites to interact with H_2O and enhance the adsorption effect, inducing the formation of a hydration layer on the surface of Cu-CuAlMo$_6$. In this wetting process, the contact between water and the surface of Cu-CAlMo$_6$ film is not only a simple physical contact, but also a wetting process generated by chemical interactions that can be proven by molecular dynamics simulations (Fig. S5). H_2O molecules in environmental humidity cannot be freely adsorbed due to the large number of confined nanopores within the Cu-CAlMo$_6$, but are restricted to form a water adsorption gradient with low inner surface water content and high outer surface water content.

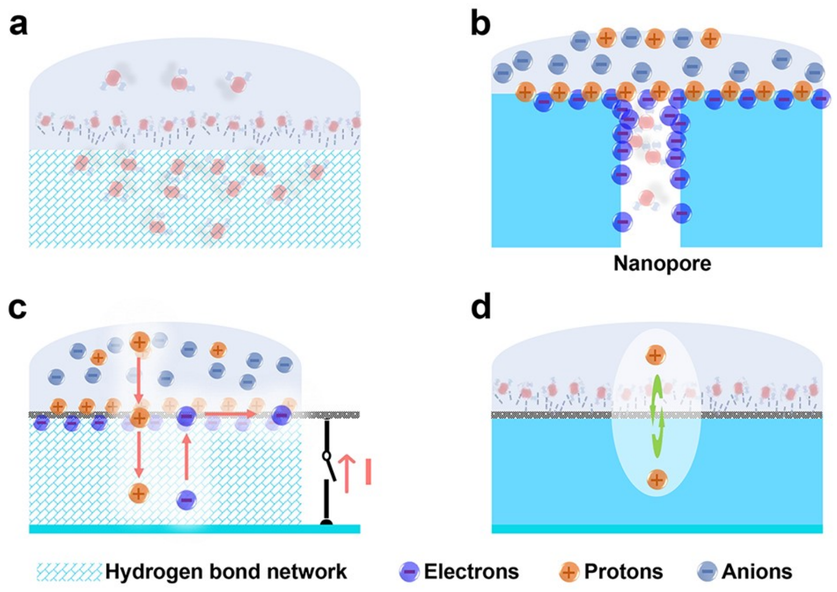

Fig. S12. Environment-powered hydrovoltaic energy conversion mechanism of Cu-CuAlMo$_6$ device.

a) The adsorption of environmental humidity and the formation of water adsorption gradient. b) The generation of EDL and charge gradient at the interface of Cu-CuAlMo$_6$/humidity. c) The interaction and the proton/charge transfer by dynamic hydrogen bond network. d) The dynamic humidity adsorption-desorption and hydrogen bonding reorganization for continuous environmental humidity power generation.

Secondly, the interfacial interaction is generated between H_2O molecules and Cu-CuAlMo$_6$ at the interface, which forms electrical double layer (EDL) structure (Fig. S12b). The hydrogen bonds and protons in water tend to transfer towards the Cu-CuAlMo$_6$ and the electrons tend to transfer from the Cu-CuAlMo$_6$ film into the water, which is influenced by the charge interactions and supported by the TPV result (Fig. 4g and 4h). The water adsorption gradient of the film leads to a difference in the amount of charge transfer between the inner and outer surfaces, resulting in a charge gradient.

Thirdly, the charge transfer process of the Cu-CuAlMo$_6$ device is realizes through the effective charge transfer platform composed of the rich hydrogen bond network in the Cu-CuAlMo$_6$ film

and water layer (Fig. S12c). The hydrogen bond network has the efficient characteristic of transferring protons and charges with the proton transfer direction of H_3O^+ to H_2O to OH^-, which is accompanied by the electron transfer in the opposite direction of the proton. The efficiency and speed of charge transfer within the Cu-$CuAlMo_6$ film are greatly improved with the rapidity and low loss characteristics of hydrogen bonds transmission. And the efficient electrical output of Cu-$CuAlMo_6$ device is completed by the coordination of external circuits.

Finally, continuous electrical output of the Cu-$CuAlMo_6$ device is generated by the dynamic humidity adsorption-desorption (Fig. S12d). The process of environmental humidity adsorbed by Cu-$CuAlMo_6$ film is a continuous adsorption-desorption equilibrium. Therefore, the environmental humidity continuously provides energy to the device by the hydrogen bonds between water molecules and Cu-$CuAlMo_6$ film. The charge is transferred with the adsorption of water molecules and the formation of EDL, thereby maintaining a sustained charge gradient within the device. The charges "pass" through the hydrogen bonding network within the Cu-$CuAlMo_6$ at a low energy barrier, low loss, and fast speed, achieving continuous environmental humidity power generation.

The rich hydrogen bonding structure endows the device with efficient water collection capability, increasing the water adsorption on the surface and promoting the formation of the double layer (Fig. S11 and S12a-b). The nanoscale pores can effectively limit the sustained adsorption of water molecules, thereby promoting a significant reduction in water adsorption capacity in the device and improving the adsorption gradient difference (Fig. S12a). The synergistic effect of both enhances the adsorption of environmental humidity and promotes the formation of water adsorption gradient, making the device compatible with high and low humidity, achieving continuous power generation in fluctuating humidity environments.

5 The ionic recognition and real-time monitoring performance of $Cu-CuAlMo_6$ humidity power generation device.

5.1 The charge structure and charge transfer property of $Cu-CuAlMo_6$ nanomaterials in ionic adsorption.

The adsorption sites of urea molecules (Fig. S13a-c) and NaCl (Na^+ and Cl^- in Fig. S13d-f) in aqueous solution environments are confirmed by the same method. The two $-NH_2$ groups of urea molecules are respectively adsorbed onto the Cl atom of the $Cu-CuAlMo_6$ material and the terminal oxygen of the adjacent polyoxoanion (Fig. S13c). This configuration facilitates more substantial electron transfer from urea to the POM framework (Fig. 5a). The Na^+ is adsorbed on the terminal oxygen atoms of polyoxoanions, and the Cl^- is adsorbed on the free H_2O molecules in the solution (Fig. S13f). This unique adsorption geometry induces charge delocalization throughout the aqueous environment (Fig.5b). The adsorbed substances and adsorption sites on the $Cu-CuAlMo_6$ are highlighted by atoms of O (red), H (white), Na (purple), Cl (green), N (gray), C (yellow). It can be observed that the adsorption behavior of various molecules by $Cu-CuAlMo_6$ mainly occurs within the nanopores (Fig. S13).

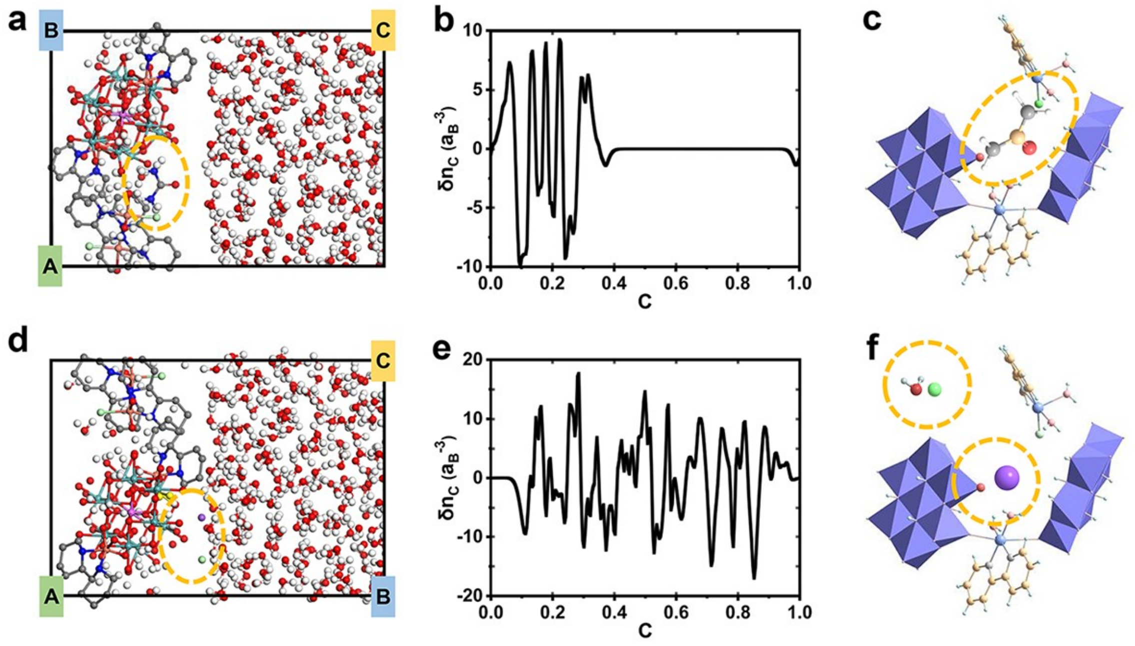

Fig. S13. The molecular dynamics simulation of ions adsorbed by Cu-CuAlMo$_6$.

a) The molecular dynamics simulation results of urea (circled by an orange dotted line) adsorbed by Cu-CuAlMo$_6$. b) The adsorption energy of adsorbed urea molecules along the C-axis direction. c) The adsorption site of adsorbed urea in aqueous solution. d) The molecular dynamics simulation results of NaCl (Na^+ in purple and Cl^- in green circled by an orange dotted line) adsorbed by Cu-CuAlMo$_6$. e) The adsorption energy of adsorbed NaCl (Na^+ and Cl^-) molecules along the C-axis direction. f) The adsorption site of adsorbed NaCl (Na^+ and Cl^-) in aqueous solution.

The charge amounts of adsorbed urea and NaCl respectively are 0.415 e (Fig. S14a) and -0.412 e (Fig. S14c), indicating that electrons transfer from H_2O and urea molecules to the Cu-CuAlMo$_6$ (Fig. S14b), and from the Cu-CuAlMo$_6$ to Na^+ (Fig. S14d). The direction and amount of charge transfer during the adsorption of different molecules by Cu-CuAlMo$_6$ are different, demonstrating flexible adsorption of chemical substances and the selective charge transfer of Cu-CuAlMo$_6$.

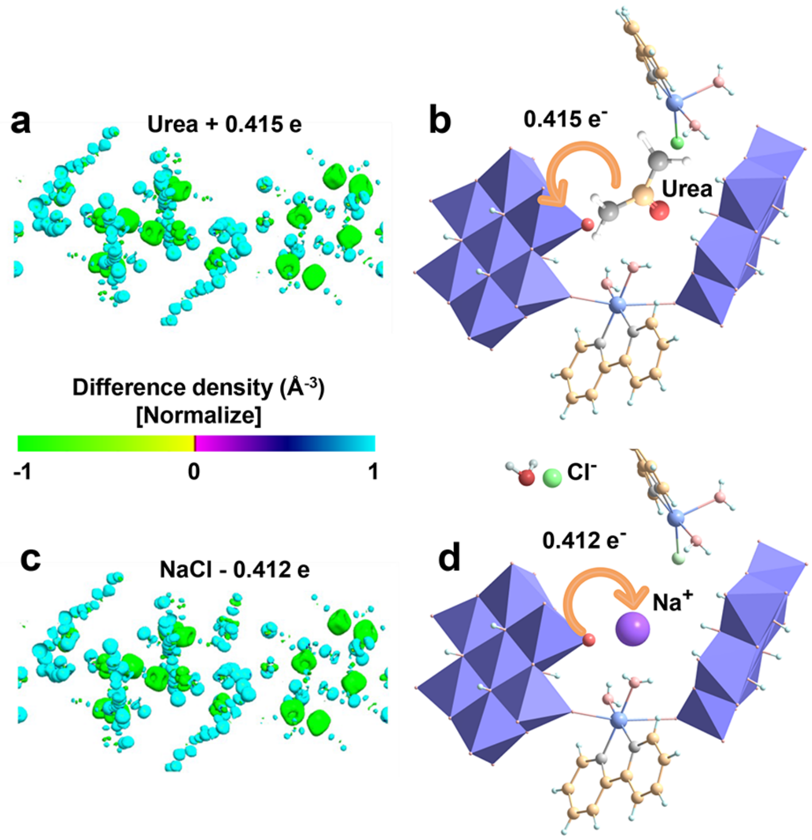

Fig. S14. Component-specific differential charge density and directional charge transfer of Cu-CuAlMo6 with adsorbate interactions.

a) The three-dimensional view of the differential charge density for urea molecule adsorbed by Cu-CuAlMo6 (color scale: normalized electron density difference). The numbers marked in the Figures represent the charge amounts of adsorbed molecules from the Bader calculation results. b) Directional charge transfer for urea, with orange arrows denoting electron flow between adsorbates and the POM framework (stick models). c) The three-dimensional view of the differential charge density for NaCl (Na^+ and Cl^-) adsorbed by Cu-CuAlMo6. d) Directional charge transfer for adsorbed NaCl.

The Difference densities in two-dimensional projection of differential charge density for Cu-CuAlMo6 with adsorbed ions (Fig. S14a and S14c) show the actual calculated quantity. The three-dimensional differential charge density (Fig. S14b and S14d) shows more intuitive charge transfer situations, with the normalized Difference density. Therefore, the two-dimensional

projection of differential charge density and three-dimensional differential charge density show different numerical representations of the same calculation result.

The differential charge density distribution shows significant difference between urea and NaCl. The urea molecule is adsorbed by the Cu-$CuAlMo_6$, with the main charge transfer occurring on the Cu-$CuAlMo_6$ molecule (Fig. S13c). The charge reconstruction occurs on the entire Cu-$CuAlMo_6$ by the unique molecular orbital structure of polyoxometalates (Fig. 5a). For the adsorption with NaCl molecules, the charge transfer and electron reconstruction on Cu-$CuAlMo_6$ are consistent with those on urea (Fig. S13f and 5b). The difference is that NaCl exist as Na^+ and Cl^- in an aqueous state, with Na^+ is adsorbed by Cu-$CuAlMo_6$, while Cl^- is adsorbed by H_2O in water environments (Fig. S13f). Therefore, as shown in the upper part of the differential charge density distribution, there is also delocalized and significant charge transfer in the water environment (Fig. 5b and S14).

5.2 The properties of Cu-$CuAlMo_6$ film with ions.

The FTIR and XRD results show that after multiple cycles of adsorption and desorption with chemical molecules for Cu-$CuAlMo_6$, the structure and crystal properties of the Cu-$CuAlMo_6$ remained stable, proving that the structure of the Cu-$CuAlMo_6$ nanomaterials has excellent chemical stability.

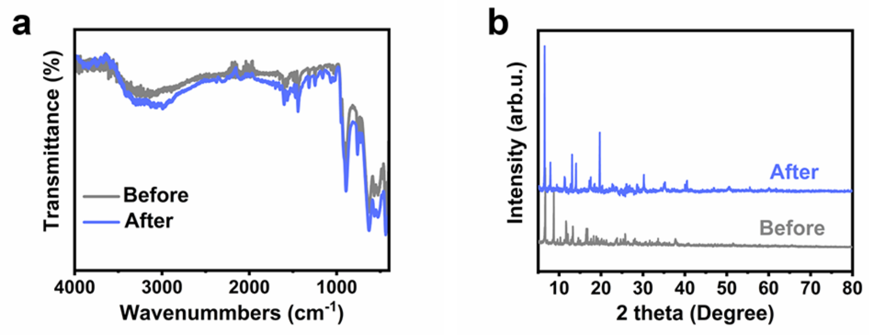

Fig. S15. The chemical stability of Cu-$CuAlMo_6$@ion film.

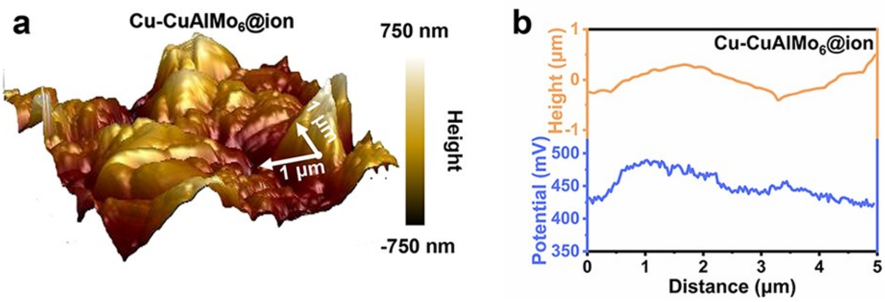

Fig. S16. KPFM analysis of Cu-$CuAlMo_6$@ion film.

a) The surface topography features of ion-incorporated Cu-$CuAlMo_6$ film. b) The surface height (orange) and surface potential (blue) with the section position at the orange line on the Cu-$CuAlMo_6$@ion film in Fig. S14a.

The XPS of Cu-CuAlMo$_6$@ion is tested by adding the solute to the Cu-CuAlMo$_6$ film with the C element as standard (Fig. S17a). The XPS of O 1s is shown in Fig. S17b. There are three kinds of O in the material: O^{2-} (530.4 eV), OH (531.7 eV), and double bond O (L=O, 532.3 eV). The two peaks at about 934.6 eV and 954.5 eV are respectively the 2P$_{3/2}$ and 2p$_{1/2}$ of Cu^{2+} (Fig. S17c). In the XPS of Cl, the two peaks at about 197.9 eV and 199.6 eV are respectively the 2P$_{3/2}$ and 2p$_{1/2}$ of Cl^- (Fig. S17d). The peaks of about 232.5 eV and 235.6 eV are respectively the 3d$_{5/2}$ and 3d$_{3/2}$ of Mo^{6+} (Fig. S17e). The peaks of Al are shown at about 77 eV (Fig. S17f). The XPS results show a shift in peak position by the addition of ion components to the Cu-CuAlMo$_6$ film (below in Fig. S167-f), indicating that there is a charge transfer between the Cu-CuAlMo$_6$ film and the ion components.

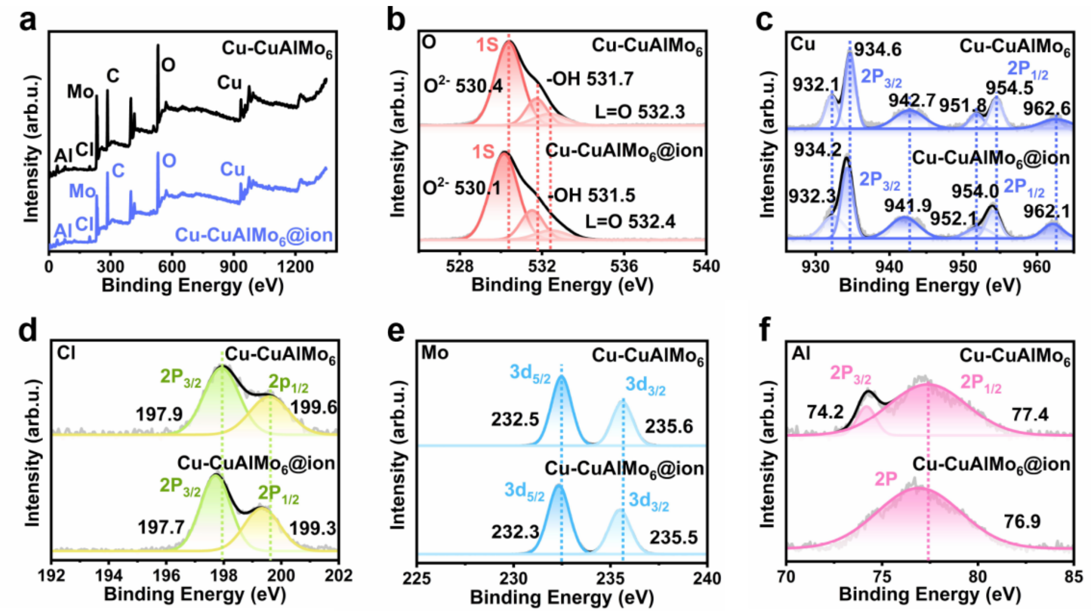

Fig. S17. XPS spectral shifts upon ion incorporation of Cu-CuAlMo$_6$ film.

a) The comparison of overall XPS result of Cu-CuAlMo$_6$ film (black) and Cu-CuAlMo$_6$ film with ions (blue). b-f) The comparison of XPS between Cu-CuAlMo$_6$ film with ions and Cu-CuAlMo$_6$ in O (b), Cu (c), Cl (d), Mo (e), an Al (f).

5.3 The environmental illumination-response of the Cu-$CuAlMo_6$ device.

The xenon lamp with optical power density of 100 mW cm^{-2} is used as the illumination source. The environment response performance of the Cu-$CuAlMo_6$ based device to illumination is tested by recording the triggered electrical signals under anhydrous with illumination and darkness. The test results show that the device exhibits a gradually increasing V_{OC} (orange in Fig. S16a) as the illumination is turned on (light orange background), and the V_{OC} of the device gradually decreases back to the starting point as the illumination is removed (light gray background) within a time interval of 100 s (50 s of illumination and 50 s of darkness). No I_{SC} signal is detected under anhydrous environment (blue curve in Fig. S16a), which indicates that the Cu-$CuAlMo_6$ based device is triggered by illumination to generate photo generated electrons, but the effective charge transfer paths are lacked, inducing that the V_{OC} can be detected but the triggered power generation unable to be achieved.

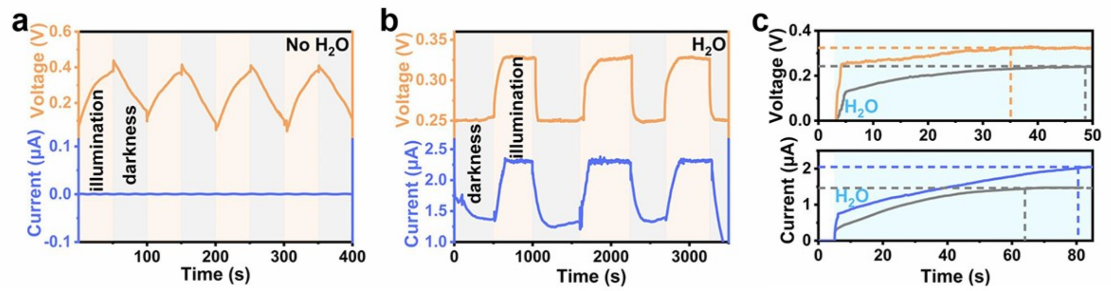

Fig. S18. Photoresponsive triggered current and voltage characteristics of Cu-$CuAlMo_6$ device. a) The triggered electrical signal of the switching illumination (the voltage in orange and the current in blue) under anhydrous environments. The illuminated and darkness environments are respectively represented by the light orange and light gray background. b) The environment response process (voltage in orange and current in blue) of the Cu-$CuAlMo_6$ device to the switching illumination in deionized water. c) The comparison of environment response process

under illuminated (the voltage in orange and the current in blue) and darkness (grey) triggered by deionized water.

The different electrical signals can be triggered by Cu-$CuAlMo_6$ devices under different light environments, which is manifested as the differences in I_{SC}, V_{OC}, t_{Isc}, t_{Voc}, $t_{90\%\ Isc}$, and $t_{90\%\ Voc}$, indicating that humidity self-power generation performance and response process of the device are influenced by illumination.

The V_{OC} (orange curve in Fig. S18b) and I_{SC} (blue curve in Fig. S18b) of the Cu-$CuAlMo_6$ device are gradually increased and stabilized at high values as the illumination is turned on, and then gradually decreased and stabilized at lower values as the illumination is disappeared (Fig. S18b). The test result shows that the triggered electrical signal of the Cu-$CuAlMo_6$ device triggered by H_2O is changed with the variation of illumination. The environment response process of the device is tested in illumination and H_2O, which shows the increased I_{SC} (blue) and V_{OC} (orange), and a shortened t_{Voc} and an extended t_{Isc} for the device (Fig. S18c and Table S5). The t_{Isc} and t_{Voc} in illumination are respectively 167% and 83% of that in darkness (gray in Fig. S18c), which shows the influence of illumination on the response processes of voltage are not consistent with that of current. In summary, the triggered electrical signals and response process of the Cu-$CuAlMo_6$ device in H_2O can be affected by the illumination, which can be used to autonomously and real-timely monitor the environmental illumination information.

Table S5. The electrical signals of the Cu-$CuAlMo_6$ device in different light environments.

Environment	I_{SC} [μA]	V_{OC} [V]	t_{Isc} [s]	$t_{90\%\ Isc}$ [s]	t_{Voc} [s]	$t_{90\%\ Voc}$ [s]
darkness	1.4452	0.2486	63.9	40.7	38.7	22.8
illumination	2.3040	0.3215	106.7	75.9	32.3	18.0

5.4 The ionic feedback-regulated electrical output of the Cu-$CuAlMo_6$ device.

The Cu-$CuAlMo_6$ devices are triggered by aqueous solutions with different concentrations for NaCl (0.5 mmol L^{-1}, 2.0 mmol L^{-1}, 10.0 mmol L^{-1}, 50.0 mmol L^{-1}, and 200.0 mmol L^{-1}) to generate different electrical signals of I_{SC}, V_{OC}, t_{Isc}, t_{Voc}, $t_{90\%\ Isc}$, and $t_{90\%\ Voc}$ (Fig. S19 and Table S6). The electrical signals show certain pattern with the change of concentrations, indicating that there are mathematical laws between the electrochemical signals and the solution concentrations. The mathematical laws of electrical signals changing with concentrations are altered under illumination, indicating that the application of illumination to the device may alter the properties of the Cu-$CuAlMo_6$ film.

The electrical signals generated by the devices Cu-$CuAlMo_6$ are also changed by the type of ions, which is based on the ionic recognition ability of Cu-$CuAlMo_6$. The electrical signals generated by the devices in different ions environments are shown in the Fig. S20-S27 (Fig. S20 for KCl, Fig. S21 for $CaCl_2$, Fig. S22 for $MgCl_2$, Fig. S23 for Na_2HPO_4, Fig. S24 for Lactic acid, Fig. S25 for Ascorbic acid, Fig. S26 for Urea,).

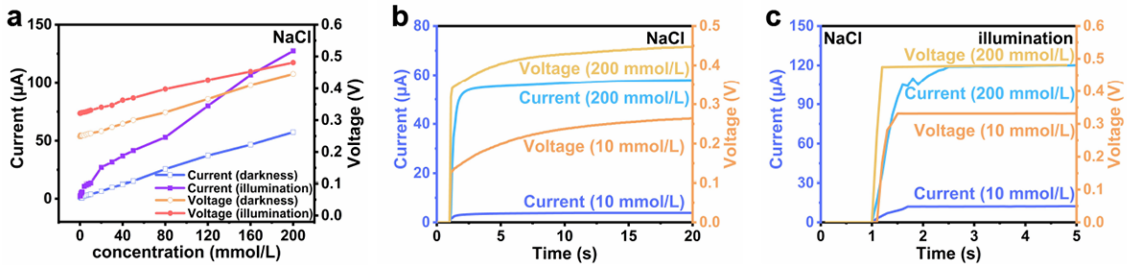

Fig. S19. Concentration- and illumination-dependent response profiles for NaCl aqueous solutions.

a) The I_{SC} and V_{OC} of the Cu-$CuAlMo_6$ device triggered by NaCl aqueous solutions with different concentrations under illumination (red for V_{OC} and purple for I_{SC}) and darkness (orange for V_{OC} and blue for I_{SC}). b) The electrical response process of the Cu-$CuAlMo_6$ device triggered by NaCl aqueous solutions with different concentrations (yellow for V_{OC} and cyan for I_{SC} at

high concentration, orange for V_{OC} and blue for I_{SC} at low concentration) under darkness. c) The electrical response process of the Cu-$CuAlMo_6$ device triggered by NaCl aqueous solutions with different concentrations (yellow for V_{OC} and cyan for I_{SC} at high concentration, orange for V_{OC} and blue for I_{SC} at low concentration) under illumination.

Table S6. Concentration- and light-dependent electrical response of Cu-$CuAlMo_6$ device for NaCl.

Environments	Concentration [mmol L^{-1}]	I_{SC} [μA]	V_{OC} [V]	t_{Isc} [s]	$t_{90\%\ Isc}$ [s]	t_{Voc} [s]	$t_{90\%\ Voc}$ [s]
darkness	0.5	1.5226	0.2505	14.2	2.5	6.3	3.8
darkness	2.0	1.7037	0.2515	9.7	0.5	4.5	1.3
darkness	10.0	3.8160	0.2587	11.1	2.8	10.9	6.2
darkness	50.0	15.324	0.3000	14.8	2.5	12.3	5.5
darkness	200.0	57.388	0.4447	14.5	1.3	6.1	2.2
illumination	0.5	2.8319	0.3219	5.0	2.5	1.3	0.6
illumination	2.0	5.9697	0.3239	9.3	2.4	0.6	0.4
illumination	10.0	13.578	0.3312	12.1	3.5	0.4	0.3
illumination	50.0	41.586	0.3698	12.9	0.8	0.7	0.2
illumination	200.0	127.39	0.4808	13.1	1.4	0.5	0.2

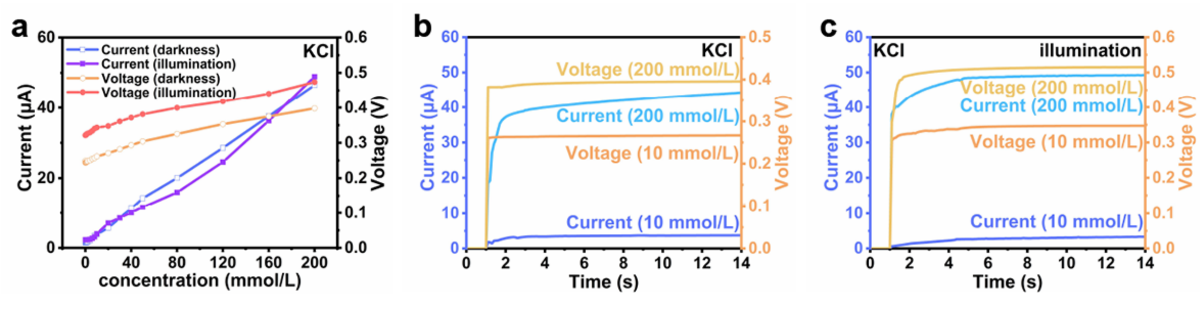

Fig. S20. Composition concentration and illumination-dependent response profiles for KCl aqueous solutions.

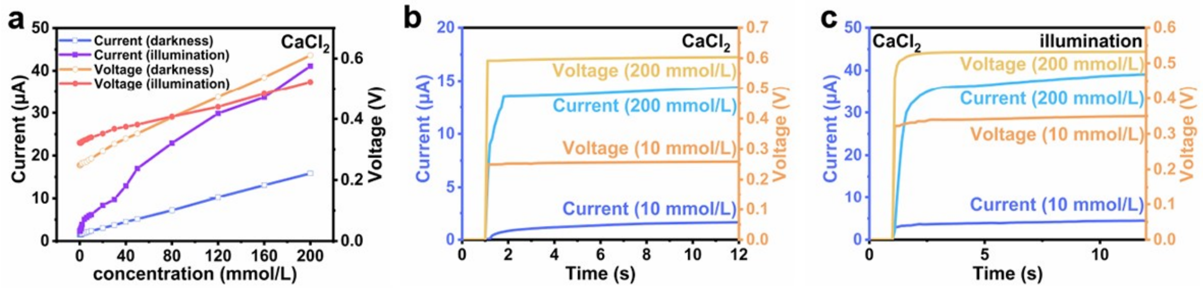

Fig. S21. Composition concentration and illumination-dependent response profiles for $CaCl_2$ aqueous solutions.

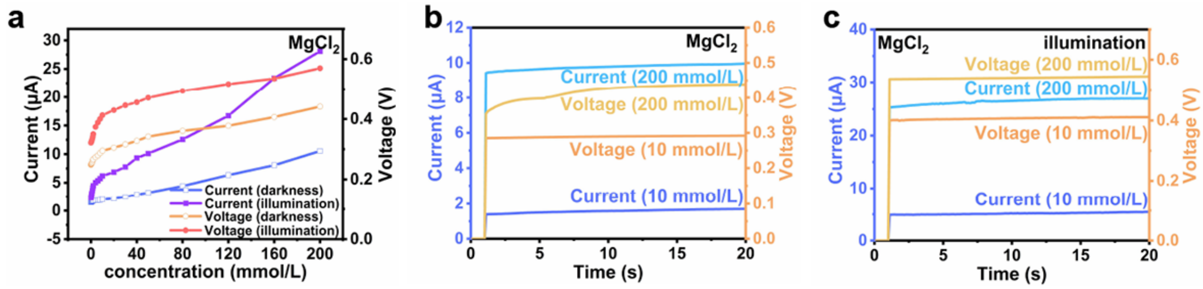

Fig. S22. Composition concentration and illumination-dependent response profiles for $MgCl_2$ aqueous solutions.

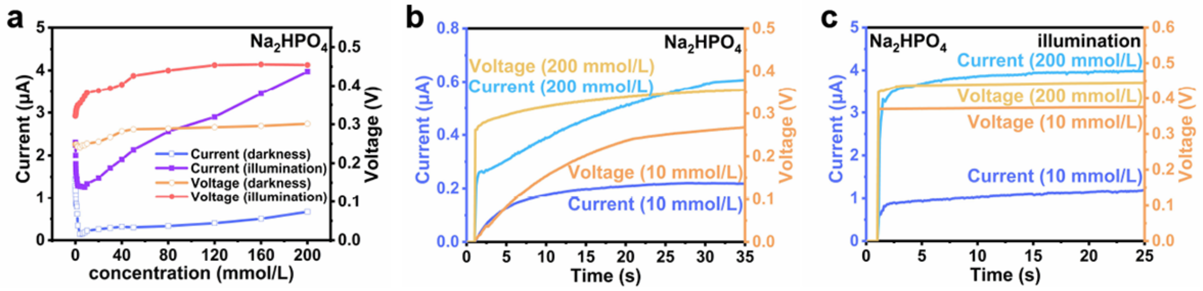

Fig. S23. Composition concentration and illumination-dependent response profiles for Na_2HPO_4 aqueous solutions.

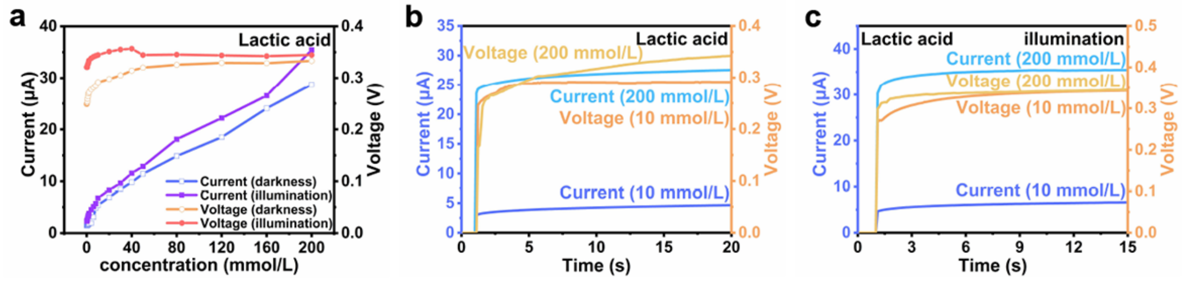

Fig. S24. Composition concentration and illumination-dependent response profiles for lactic acid aqueous solutions.

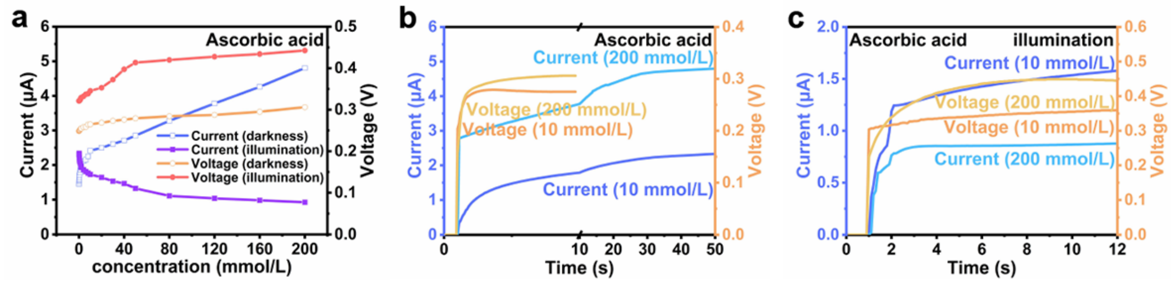

Fig. S25. Composition concentration and illumination-dependent response profiles for ascorbic acid aqueous solutions.

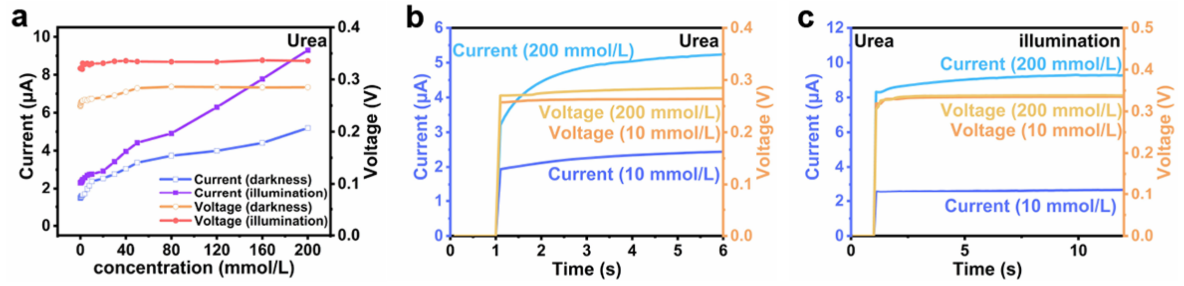

Fig. S26. Composition concentration and illumination-dependent response profiles for urea aqueous solutions.

The Cu-CuAlMo$_6$ devices are triggered by aqueous solutions with different components at the same concentration to generate the different electrical signals of I_{SC}, V_{OC}, t_{Isc}, t_{Voc}, $t_{90\%\ Isc}$, and $t_{90\%\ Voc}$, indicating that the devices are greatly influenced by the type of ions in the solution (Fig. S25, Table S7 and S8). The Cu-CuAlMo$_6$ device has the potential to identify the component

and concentration of multiple ions. The Fig. S27c-d and Table S8 shows significant differences in I_{SC}, V_{OC}, t_{Isc}, t_{Voc}, $t_{90\%\ Isc}$, and $t_{90\%\ Voc}$ under illumination. The component and concentration of ions in the solution under illumination have different effects on the device compared to that under darkness, which is beneficial for improving the detection sensitivity of the device by analyzing the difference in electrical signals between the two states of illumination and darkness.

The value and response time of I_{SC} and V_{OC} are regular change with the concentrations for the same solute. And different I_{SC} and V_{OC} are generated by the device in the solutions with the same concentration but different ions. The times required for the maximum voltage and current of the device are different, which proves that the device in the solutions with different solutes shows differentiate electrochemical response speed.

According to the above tests (Fig. S19-S27, Tables S6-S8), the differences of solutions in solutes and concentrations can be displayed through the values and response times of electrical signals, as the device is placed in different solution environments. The electrical signals excited by the same solution are changed by changing the lighting conditions, which is because that the ions in the solution can interact with the Cu-$CuAlMo_6$ film. The electronic structure of Cu-$CuAlMo_6$ material and the interaction mode between each ion and the Cu-$CuAlMo_6$ film might be changed by illumination. The synergistic combination of concentration linearity, composition selectivity, and environmental modulation establishes a versatile platform for self-powered real-time monitoring of environment.

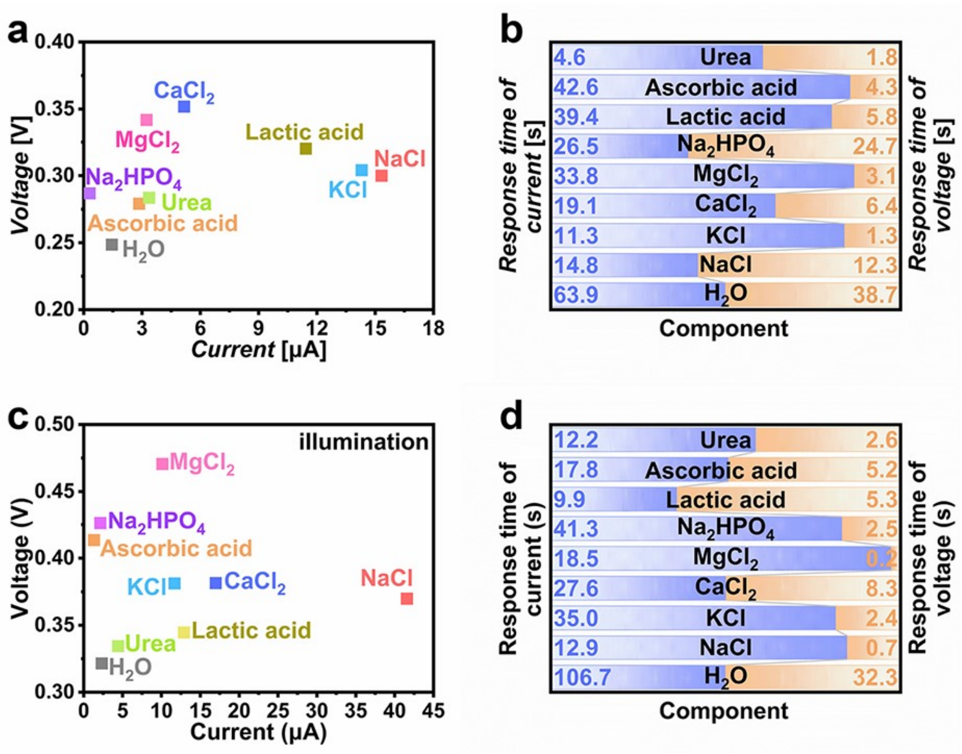

Fig. S27. Composition and concentration dependent response profiles of Cu-$CuAlMo_6$ devices. a) The V_{OC} and I_{SC} (blue) generated by Cu-$CuAlMo_6$ devices in different compositions of H_2O (grey), NaCl (red), KCl (cyan), $CaCl_2$ (blue), $MgCl_2$ (pink), Na_2HPO_4 (purple), lactic acid (orange), ascorbic acid (yellow), and urea (green). b) The t_{Isc} (blue) and t_{Voc} (orange) of the Cu-$CuAlMo_6$ device in different compositions. c) The V_{OC} and I_{SC} (blue) generated by Cu-$CuAlMo_6$ devices in different compositions under illumination. d) The t_{Isc} (blue) and t_{Voc} (orange) in different compositions under illumination.

Table S7. Composition-specific electrical signatures for 200 mM solutions in darkness.

Component	I_{SC} [μA]	V_{OC} [V]	t_{Isc} [s]	$t_{90\%\ Isc}$ [s]	t_{Voc} [s]	$t_{90\%\ Voc}$ [s]
NaCl	57.386	0.4421	12.86	1.92	8.02	3.8
KCl	46.556	0.3481	15.14	3.72	1.94	0.22
$CaCl_2$	15.834	0.6022	18.00	5.66	6.94	0.24
$MgCl_2$	10.427	0.4575	31.84	7.92	2.68	0.16
Na_2HPO_4	0.8566	0.3182	29.34	14.86	24.12	11.24
Lactic acid	28.721	0.3332	40.44	15.4	6.84	1.68
Ascorbic acid	4.1492	0.3182	40.40	16.54	4.76	1.36
Urea	5.6901	0.2682	4.44	1.4	2.3	0.276

Table S8. Composition-specific electrical signatures for 200 mM solutions in illumination.

Component	I_{SC} [μA]	V_{OC} [V]	t_{Isc} [s]	$t_{90\%\ Isc}$ [s]	t_{Voc} [s]	$t_{90\%\ Voc}$ [s]
NaCl	127.39	0.4808	10.48	2.12	0.7	0.34
KCl	49.029	0.4374	33.76	20.86	2.24	0.18
$CaCl_2$	40.63	0.5341	28.58	15.06	6.98	0.36
$MgCl_2$	27.067	0.5857	16.92	1.28	0.18	0.118
Na_2HPO_4	3.9753	0.4522	36.84	13.38	1.046	0.136
Lactic acid	34.734	0.3248	11.7	3.06	5.64	0.566
Ascorbic acid	0.8528	0.4717	18.2	7.14	6.48	1.92
Urea	9.495	0.3366	11.6	0.74	2.04	0.154

As there are ionic components in the environment, firstly, the ionic components are adsorbed by POMs and undergo charge transfer (Fig. 5c). Due to the selective charge transfer performance of POM, ions are recognized. Furthermore, the adsorption and charge transfer of ions will alter the work function of the POM film as shown in the Fig. 5d, changing the interface

charge transfer properties of the device and generating a charge gradient influenced by ions (Fig. S12b). Secondly, ions can act as a part of the electrolyte, affecting the formation process of the EDL and altering the efficiency of energy conversion (Fig. S11 and S12b). Finally, ions can serve as charge transfer carriers, promoting the dynamic process of charge transfer and generating electrical signal feedback influenced by ions (Fig. S12c). As shown above, ions are adsorbed and recognized by POM from the selective charge transfer properties, changing the humidity electrical signals of the device, and further affecting the transmission process of electrical signals to achieve feedback regulation of electrical signals (Fig. S28).

The electrical signals generated in the above aqueous solution are similar to the power generation performance of the device in high humidity with condensed water, proving that the ion recognition of electrical signals is starting from the humidity power generation (the blue arrow on the left of Fig. S28). Ions are adsorbed by Cu-CuAlMo$_6$ and undergo charge transfer, resulting in changes in the water adsorption gradient and charge gradient of the devices, generating modulated humidity electrical signals. Next, the modulated humidity electrical signals are further affected by ions during transmission, resulting in further refinement of electrical signals (the cyan arrow on the left of Fig. S28). Therefore, the device changes from the adsorption equilibrium of H_2O and Cu-CuAlMo$_6$ to the interaction equilibrium of ions--H_2O--Cu-CuAlMo$_6$, and then achieves ion feedback through electrical signals (the green arrow on the left of Fig. S28). Unlike traditional devices that use fixed electrical signals for electrochemical detection, the equilibrium of ions--H_2O--Cu-CuAlMo$_6$ interactions results in the much higher amount of information and accuracy of the electrical signals that generated from ordinary non-feedback regulation devices. Ionic feedback enhances selectivity and anti-interference ability, enabling robust multi-component analysis capability in complex media of device. Continuous, real-time monitoring is achieved through the persistent electrical output, with additional analytical dimensions provided by comparative illumination/darkness response profiling. The the POM-devices generate electrical signals from external environments

(including humidity and water, sun, ions), and achieve real-time monitoring by interacting with the external environments and feedback adjusting the electrical signals.

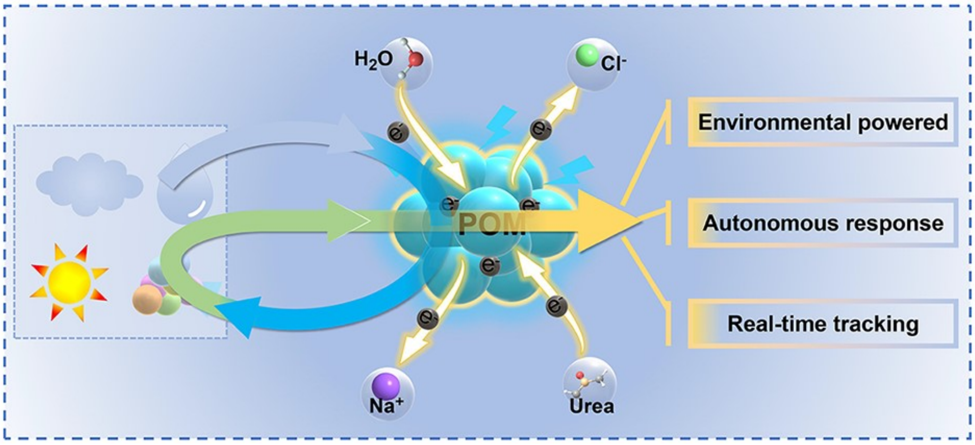

Fig. S28. The feedback regulation of Cu-$CuAlMo_6$ device proposed in this work.

Electrochemical impedance spectroscopy (EIS) analyses reveal critical insights into the interfacial processes between the Cu-$CuAlMo_6$ film and ions. The Nyquist plots demonstrate comparable series impedance across different test solutions (Fig. S29a), dominated by the intrinsic device characteristics and measurement system, while subtle variations reflect component-specific charge transfer interactions. Notably, the high-frequency region (Fig. S29b) exhibits minimal interfacial charge transfer resistance, as evidenced by the absence of distinct semicircular features, which is attributed to the highly efficient hydrogen-bond-mediated charge conduction channels established between the POM framework and aqueous phase. The subtle variations reflects component-modulated interfacial charge transfer (Fig. S29a and b). This conclusion is further corroborated by PL and TPV analyses, collectively confirming the synergistic role of hydrogen bonding in facilitating rapid charge transport. Bode phase analysis (Fig. S29c) reveals nearly identical maximum phase values across all tested systems, indicating similar magnitudes of interfacial charge transfer resistance. The slight frequency shifts at maximum phase points, indicating that electron lifetime and transfer kinetics are regulated by ions. The EIS analyses results are consistent with the demonstrated surface

charge distribution and work function influenced by ion interactions. These EIS findings provide direct electrochemical evidence for the proposed hydrogen-bond-assisted charge transfer model, while elucidating how selective ion-POM interactions fine-tune the interfacial energetics without compromising overall charge transport efficiency.

Firstly, as shown by the differential charge density results, selective charge transfer occurs as ions are adsorbed by Cu-CuAlMo$_6$, exhibiting the ionic specificity in number and direction of electron transfer (Fig. 5a and 5b). Secondly, the KPFM results demonstrate that the selective charge transfer process can change the work function of Cu-CuAlMo$_6$ (Fig. 5d), resulting in a changed EDL at the interface of Cu-CuAlMo$_6$ and water regulated by ion composition. Therefore, the ions lead to changes in the electron transfer properties and power generation capacity of the Cu-CuAlMo$_6$ film, affecting electrical signal output. Moreover, ionic components can participate as electrolyte components in the generation of the EDL, forming an enhanced EDL structure. Finally, electrochemical impedance spectroscopy (EIS) analysis revealed the interface charge transfer between Cu-CuAlMo$_6$ and the adsorbate ions, indicating that the electron transfer kinetics are ion regulated (Fig. S29). The environmental ion components can affect and participate in the charge transfer process of the Cu-CuAlMo$_6$ device.

The above results indicate that the Cu-CuAlMo$_6$ device achieves specific recognition and electrical signal feedback of ion components through selective charge transfer between ions and Cu-CuAlMo$_6$ molecules, the affected work function and power generation process, and the interface charge transfer, and has an ion recognition feedback mechanism (Fig. S28). The ion recognition feedback process can improve the selectivity and anti-interference ability of monitoring, and has strong multi-component analysis capability in complex system.

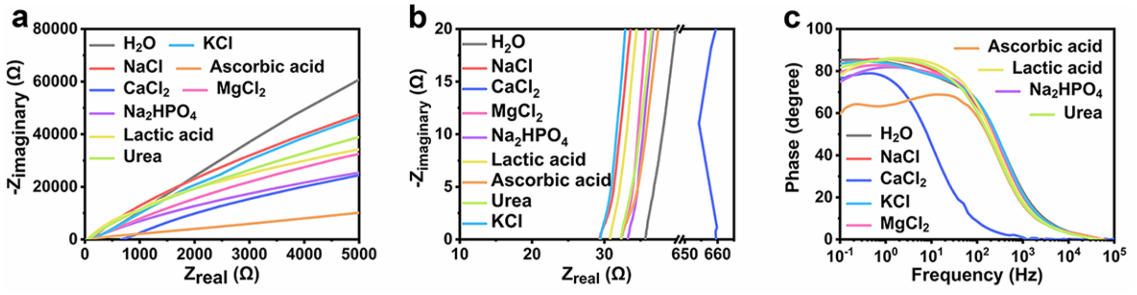

Fig. S29. The Nyquist with high frequency region of the Cu-CuAlMo$_6$ device in the aqueous solutions with different components.

In artificial sweat with altered compositions (Table S9), the device exhibits significant variations in I_{SC}, V_{OC}, (Fig. S30a), along with response time parameters (t_{Isc}, and t_{Voc}, Fig. S30b), demonstrating robust compositional sensitivity that is further modulated under illumination (Fig. S30c and S30d). The results demonstrate that the device can exhibit high-precision multi-component synchronous monitoring capability in actual complex solution environments. The applicability of the device's multi-component synchronous monitoring capability is wide. In high-concentration mixed solutions (Table S10), while I_{SC} and V_{OC} remain responsive to compositional changes (Fig. S31a and S31c), the similar t_{Isc} and t_{Voc} values (Fig. S31b and S31d) suggest that the devices have diminished component discrimination capability and are only used in humidity power generation.

Table S9. Compositional formulations of altered artificial sweat.

Different Component	NaCl [mmol L^{-1}]	KCl [mmol L^{-1}]	$CaCl_2$ [mmol L^{-1}]	$MgCl_2$ [mmol L^{-1}]	Na_2HPO_4 [mmol L^{-1}]	Lactic acid [mmol L^{-1}]	Ascorbic acid [mmol L^{-1}]	Urea [mmol L^{-1}]
NaCl	80.0	9.0	5.0	0.6	10.0	13.5	0.43	23.1
KCl	40.0	49.0	5.0	0.6	10.0	13.5	0.43	23.1
$CaCl_2$	40.0	9.0	45.0	0.6	10.0	13.5	0.43	23.1
$MgCl_2$	40.0	9.0	5.0	40.6	10.0	13.5	0.43	23.1
Na_2HPO_4	40.0	9.0	5.0	0.6	50.0	13.5	0.43	23.1
Lactic acid	40.0	9.0	5.0	0.6	10.0	53.5	0.43	23.1
Ascorbic acid	40.0	9.0	5.0	0.6	10.0	13.5	40.43	23.1
Urea	40.0	9.0	5.0	0.6	10.0	13.5	0.43	63.1

The mixed multi-component analysis capability of Cu-$CuAlMo_6$ device.

Table S10. Preparation protocols for high-concentration mixed solutions

Different Component	NaCl [mmol L^{-1}]	KCl [mmol L^{-1}]	$CaCl_2$ [mmol L^{-1}]	$MgCl_2$ [mmol L^{-1}]	Na_2HPO_4 [mmol L^{-1}]	Lactic acid [mmol L^{-1}]	Ascorbic acid [mmol L^{-1}]	Urea [mmol L^{-1}]
NaCl	80.0	40.0	40.0	40.0	40.0	40.0	40.0	40.0
KCl	40.0	80.0	40.0	40.0	40.0	40.0	40.0	40.0
$CaCl_2$	40.0	40.0	80.0	40.0	40.0	40.0	40.0	40.0
$MgCl_2$	40.0	40.0	40.0	80.0	40.0	40.0	40.0	40.0
Na_2HPO_4	40.0	40.0	40.0	40.0	80.0	40.0	40.0	40.0
Lactic acid	40.0	40.0	40.0	40.0	40.0	80.0	40.0	40.0
Ascorbic acid	40.0	40.0	40.0	40.0	40.0	40.0	80.0	40.0
Urea	40.0	40.0	40.0	40.0	40.0	40.0	40.0	80.0

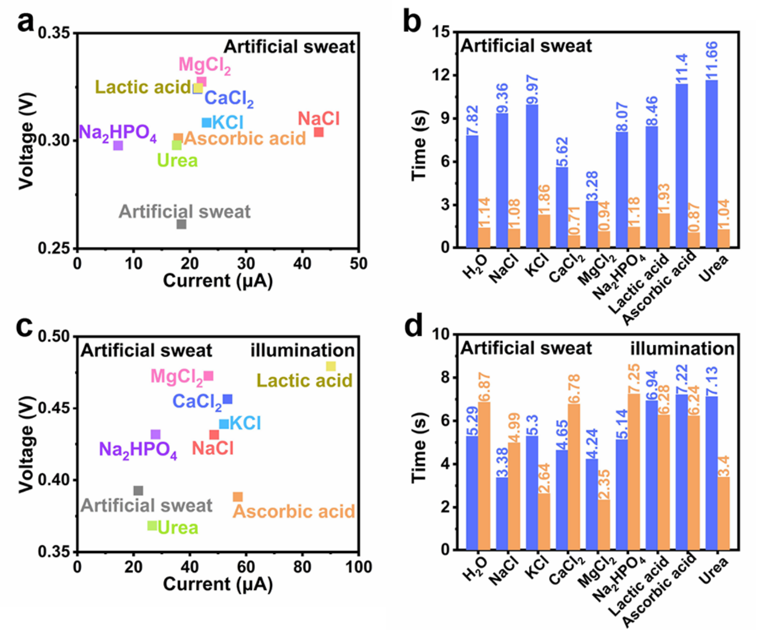

Fig. S30. Artificial sweat analysis under illumination/darkness.

a) The I_{SC} and V_{OC} of the Cu-CuAlMo$_6$ device triggered by the artificial sweats with different components under darkness. b) The t_{Isc} (blue) and t_{Voc} (orange) of the Cu-CuAlMo$_6$ device triggered by the artificial sweats with different components under darkness. c) The I_{SC} and V_{OC} under illumination. d) The t_{Isc} (blue) and t_{Voc} (orange) under illumination.

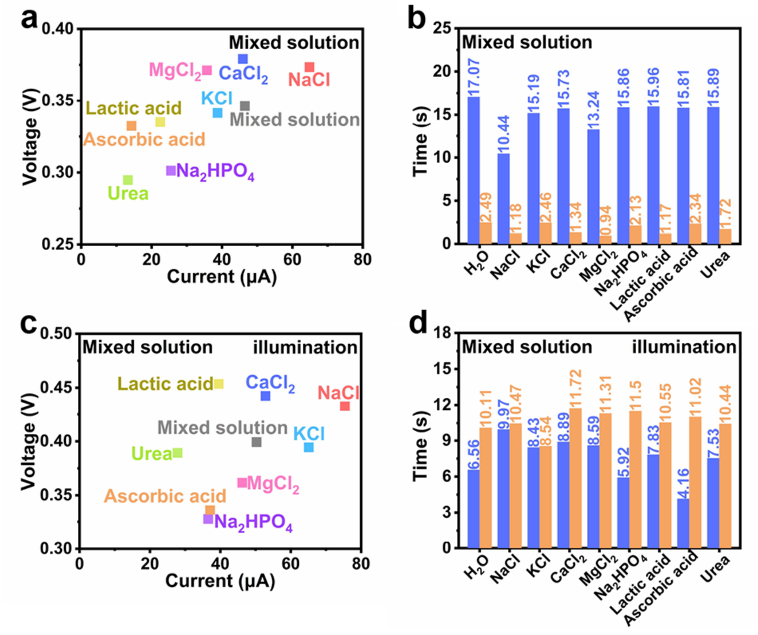

Fig. S31. High-concentration mixed solution analysis under illumination/darkness.

The differences between these EIS results show that changes in a small amount of solute in the artificial sweat can affect the EIS results of the Cu-$CuAlMo_6$ device, as changes in composition can affect the charge transfer process of the device (Fig. S32).

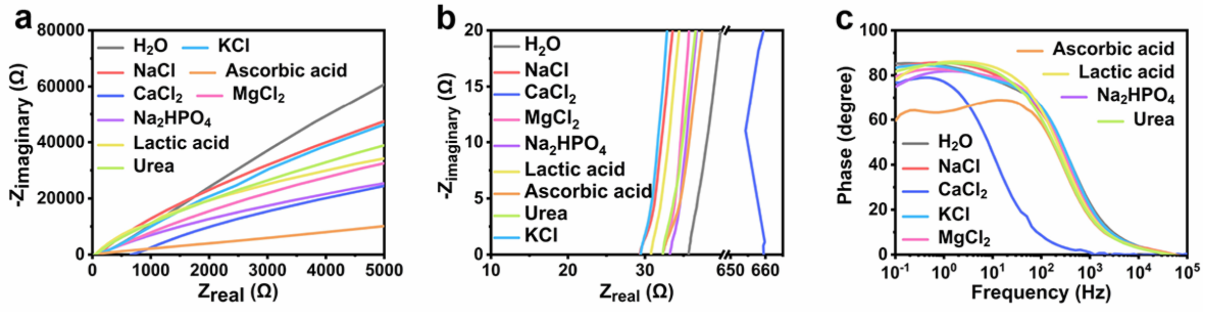

Fig. S32. EIS characterization in altered artificial sweat.

a-c) The Nyquist diagram (a), the high frequency region of Nyquist diagram (b) and Bode diagram (c) of the Cu-$CuAlMo_6$ device in artificial sweat (black curve), and artificial sweat with different components of NaCl (red), KCl (cyan), $CaCl_2$ (blue), $MgCl_2$ (pink), Na_2HPO_4 (purple), lactic acid (orange), ascorbic acid (yellow), and urea (green).

5.5 The mechanism of Cu-CuAlMo6 device for mixed multi-component monitoring.

The Cu-CuAlMo6 is represented as a film composed of blue rectangular prisms and blue spheres with nanopores represented by yellow tubes, where H_2O, urea, Na^+ and Cl^- (O in red, H in white, N in blue, C in grey, Na in purple, and Cl in green) are adsorbed into the nanopores of the Cu-CuAlMo6 (Fig. S33). The arrows in orange indicate the direction of electron transfer. The Cu-CuAlMo6 can act as donors and acceptors of electrons, which generates electrical signals and responds to various adsorbed ions.

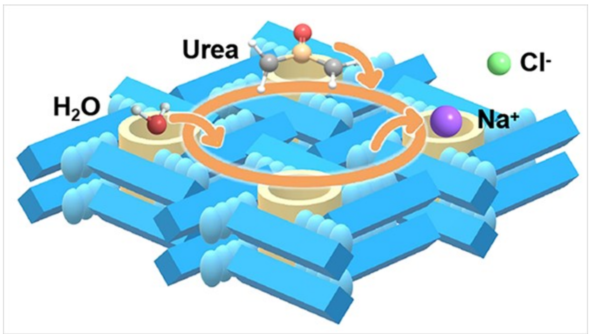

Fig. S33. Selective electron transfer pathways at ions-POM.

The proton-charge conduction process dominated by hydrogen bonds inside the Cu-CuAlMo6 film is represented by the circular arrow on the left. The proton-charge conduction process influenced by ions in the solution at the interface between the Cu-CuAlMo6 film and aqueous solution is represented by the circular arrow on the right. The balls in different colors are used to represent the different components in the solution (Fig. S34).

The Cu-CuAlMo6 device is triggered by external environment (humidity) to generate electrical signals, and achieves the real-time multi-component monitoring by the changing triggered electrical signals through ionic feedback regulation. The illumination can alter the charge structure of Cu-CuAlMo6 (the charge transfer dominated by hydrogen bonds on the left side of the Fig. S34) and the electrochemical behavior at the interface of Cu-CuAlMo6 film. The

selective electron transfer between the ions and Cu-$CuAlMo_6$ alters the electrochemical behavior of the Cu-$CuAlMo_6$ device, enabling the device to exhibit a specific environment response and ionic feedback regulation process (the charge transfer between the film and ions on the right side of the Fig. S34). Therefore, the Cu-$CuAlMo_6$ device can use external environment as energy sources to achieve the real-time multi-component autonomous analysis of complex external environments through the triggered continuous electrical signal generated by ionic feedback regulation.

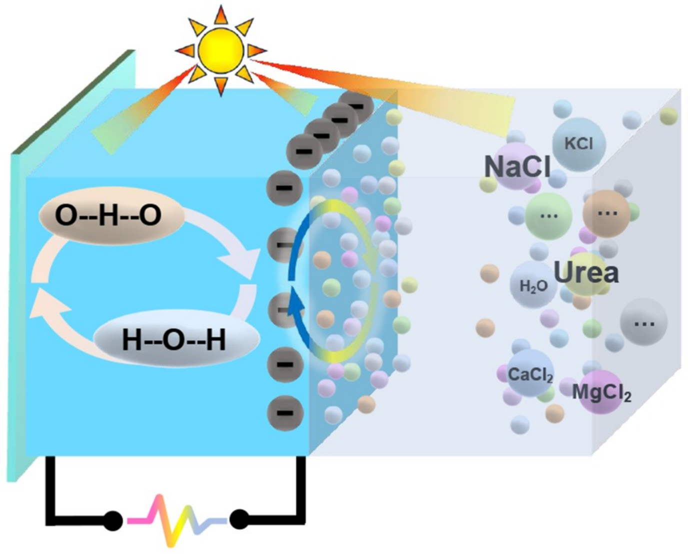

Fig. S34. The schematic diagram for the environment-powered electrochemical behavior of Cu-$CuAlMo_6$ device.

5.6 The machine learning-assisted analysis of electrical signals generated by Cu-CuAlMo$_6$ devices.

The transition of color from blue to red represents the increase in correlation (Fig. S35).

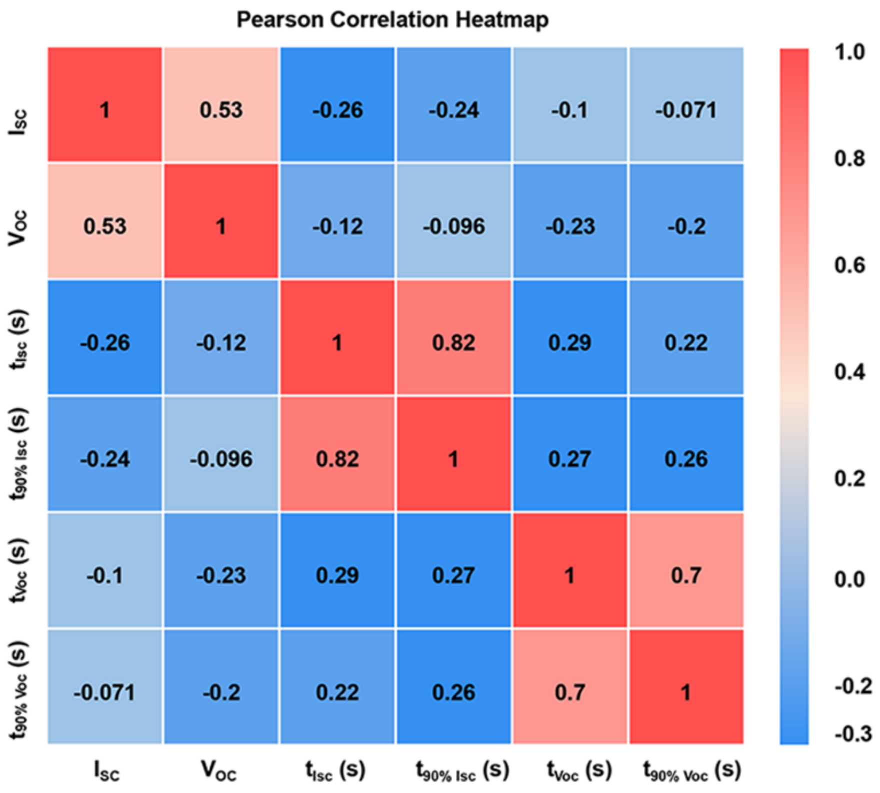

Fig. S35. The Pearson correlation analysis of triggered electrical signals.

6. The feasibility analysis of Cu-$CuAlMo_6$ device in practical application.

The experimental results show that the device achieve a stable V_{OC} of 0.265 V, I_{SC} of 171 μA cm^{-2}, and self-generated power density of 10 μW cm^{-2} in artificial sweat, which is much higher that at 100% humidity. This is closely related to the ionic recognition feedback mechanism of Cu-$CuAlMo_6$ humidity power generation device. The various ion components in sweat can undergo charge transfer with Cu-$CuAlMo_6$, reducing the work function as shown in the Fig. 5e, giving the Cu-$CuAlMo_6$ film a higher surface charge density and charge transfer ability, greatly enhancing its power generation capacity. The ion components in sweat can serve as electrolyte components to enhance the EDL and promote the charge transfer at the interface. Taking into account the above factors, the Cu-$CuAlMo_6$ device demonstrates a much higher power generation capacity in artificial sweat compared to in 100% humidity.

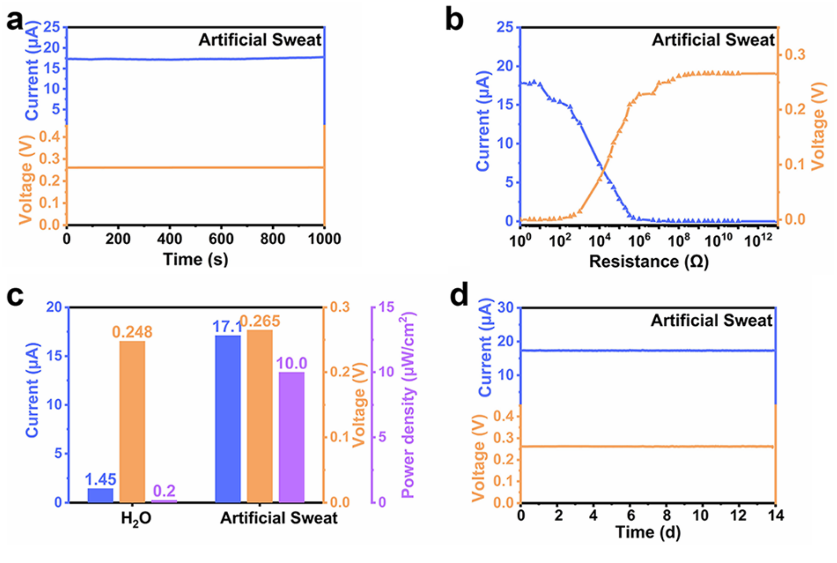

Fig. S36. The power generation performance of devices Cu-$CuAlMo_6$ in artificial sweat.

a) The stable current (blue) and voltage (orange) of the Cu-$CuAlMo_6$ device triggered by the artificial sweat. b) Current (blue) and voltage (orange) output load resistance characteristics under artificial sweat. c) The comparison on electrochemical performance of Cu-$CuAlMo_6$ device triggered by deionized water and artificial sweat (I_{SC} in blue, V_{OC} in orange and power

density in purple). d) The continuous curve (blue) and voltage (orange) for 14 days of the Cu-$CuAlMo_6$ device triggered by artificial sweat.

As shown in Figure S19-S27, in single component solution, the device can make accurate judgments and generate significantly changed electrical signals at an ion concentration of 0.1 mmol L^{-1}. In a high concentration mixed solution, taking AS solution as an example, the total component concentration can reach up to 80 mmol L^{-1}. For a concentration change of 4 mmol L^{-1}, the device can accurately identify the ion composition and generate significant electrical signal feedback (Fig. S37). The detection limit of the device can reach 0.1 mmol L^{-1} for single ion component, and for complex mixed systems with high concentrations, a 5% change in ion composition can be accurately identified and feedback.

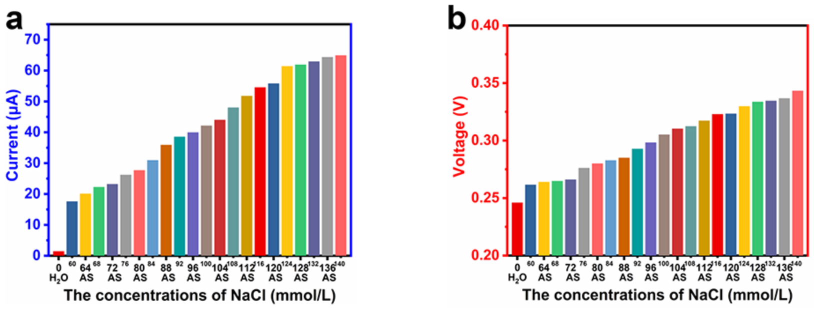

Fig. S37. The detection limit of Cu-$CuAlMo_6$ devices.

a-b) The detection and analysis current (a) and voltage (b) results of component changes in mixed systems.

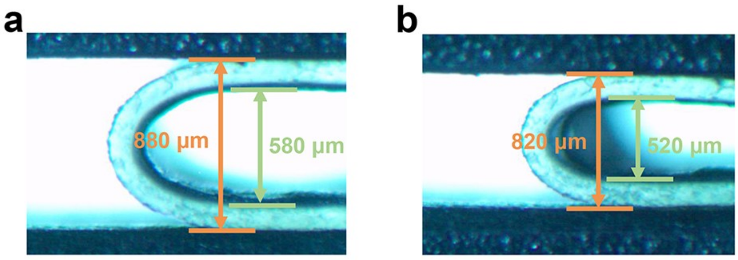

Fig. S38. The mechanical properties of Cu-$CuAlMo_6$ device.

a) The bending radius of the Cu-$CuAlMo_6$ device with 90% of its humidity self-generation performance and ionic recognition ability. b) The bending radius of the Cu-$CuAlMo_6$ device with 70% of its performance.
